# A distinct brain pathway links viral RNA exposure to sickness behavior

**DOI:** 10.1038/srep29885

**Published:** 2016-07-20

**Authors:** Xinxia Zhu, Pete R. Levasseur, Katherine A. Michaelis, Kevin G. Burfeind, Daniel L. Marks

**Affiliations:** 1 Papé Family Pediatric Research Institute, Oregon Health & Science University, Portland, OR 97239, USA; 2 MD/PhD Program, Oregon Health & Science University, Portland, OR 97239, USA

## Abstract

Sickness behaviors and metabolic responses to invading pathogens are common to nearly all types of infection. These responses evolved to provide short-term benefit to the host to ward off infection, but impact on quality of life, and when prolonged lead to neurodegeneration, depression, and cachexia. Among the major infectious agents, viruses most frequently enter the brain, resulting in profound neuroinflammation. We sought to define the unique features of the inflammatory response in the brain to these infections. We demonstrate that the molecular pathway defining the central response to dsRNA is distinct from that found in the periphery. The behavioral and physical response to the dsRNA mimetic poly I:C is dependent on signaling via MyD88 when it is delivered centrally, whereas this response is mediated via the TRIF pathway when delivered peripherally. We also define the likely cellular candidates for this MyD88-dependent step. These findings suggest that symptom management is possible without ameliorating protective antiviral immune responses.

Sickness behaviors and the metabolic responses to invading pathogens (i.e. the illness response) are common to nearly all types of infection. Fever, anorexia, lethargy, and catabolism of lean body tissues all evolved to provide short-term benefit to the host to ward off infection^1^. However, these symptoms have a tremendous impact on quality of life, and when prolonged can lead to neurodegeneration, depression, and cachexia^2^,^3^. Among the major infectious agents, viruses most frequently enter the brain and simultaneously employ myriad strategies for maintaining chronic infection, often resulting in profound morbidity and mortality secondary to neuroinflammation^4^. A number of viral pathogen associate molecular patterns (PAMPs) are recognized by the innate immune system, including dsRNA, which is an intermediate in the replicative cycle of all virus types except negative strand RNA viruses^5^. In peripheral tissues, the primary signaling pathway for the immune response to dsRNA involves binding to Toll-Like Receptor 3 (TLR3), which transduces its signal primarily via TIR-domain-containing adapter-inducing interferon-β (TRIF), unlike most other TLRs and the type 1 interleukin 1-receptor (IL-1R1) that signal primarily via myeloid differentiation primary response gene 88 (MyD88)^6^. In some cases, an alternative dsRNA-sensing pathway is engaged that utilizes the RNA helicases retinoic acid-inducible gene-1 (RIG-1) and melanoma differentiation-associated protein-5 (MDA5), both requiring interferon beta promoter stimulator 1 (IPS-1) as an obligate signaling partner^7^. Regardless of the signaling pathway, it is clear that dsRNA is a common and robust PAMP for initiation of antiviral immune activation in peripheral cells. In contrast, relatively little is known about the unique features of the immune response in the brain to dsRNA, and the central mechanisms linking viral infections to the illness response remain undefined. It is also clear that maternal exposure to viral PAMPs leads to unique innate immune responses in the fetal brain that are accompanied by long-term behavioral alterations in the offspring, further emphasizing the need to understand this signaling pathway in the brain^8^,^9^. In this paper we present evidence *in vivo* that the CNS senses the dsRNA mimetic polyinosinic:polycytidylic acid (poly I:C) by a mechanism that is distinct from that found in the periphery, independent of signaling via TLR3, TRIF, or IPS-1, but dependent on MyD88.

In the absence of immune challenge or tissue damage, the primary function of microglia is to provide a supportive environment for neuronal function by clearing toxic cellular waste, releasing various anti-inflammatory and neurotrophic factors, and pruning neuronal synaptic connections^10^. However, during the acute phase of viral infection, microglia also produce an array of cytokines, interferons, and chemokines to directly inhibit viral function, and direct immune traffic into the brain. Because viruses are intracellular pathogens, microglia must also function as antigen-presenting cells to coordinate and activate recruited T-cells^4^,^11^. Thus, from an immunological perspective, microglia in a healthy CNS exist in a quiescent state, but become highly activated in response to CNS injury or infection with various pathogens, including many types of virus^12^. To some extent, these two critical roles of microglia are mutually exclusive, and chronic microglial activation by viruses or other toxic insults therefore results in neuronal dysfunction and neurodegeneration^2^,^12^. Microglia have a unique developmental ontogeny that is distinct from both neurons and macroglia. Recent studies demonstrate that microglia originate from a primitive macrophage pool in the embryonic yolk sac and directly invade the brain at early developmental stages (reviewed in ref. 11). These are joined at later developmental stages by marrow-derived perivascular macrophages and dendritic cells that occupy unique niches in the brain that have little overlap with the brain parenchyma (e.g. meninges, choroid plexus)^13^,^14^. This provides a clear rationale for re-evaluating the basic signaling mechanisms underlying microglial immune responses, their interaction with macroglia (e.g. astrocytes) and their role in the link between infection and sickness behaviors. The *in vitro* studies presented here demonstrate that a key subset of proinflammatory cytokines is induced by poly I:C in both microglia and astrocytes via a unique MyD88-dependent pathway.

Although many brain areas respond to the inflammation produced by infectious pathogens, it is clear that the medial-basal hypothalamus (MBH) is a critical brain region for both sensing and amplifying these signals^15^,^16^,^17^. Indeed, a common early event in a variety of illness states is the induction and release of IL-1β within the MBH, thereby providing a durable paracrine inflammatory signal within this brain region^18^,^19^. A number of neuronal populations, including MBH pro-opiomelanocortin (POMC) neurons, respond to these local inflammatory signals and play a key role in transducing inflammatory signals into sickness behaviors^20^,^21^. However, despite the prevalence and severity of the illness response, there is no consensus on the mechanisms whereby viral infections are sensed and translated into alterations in hypothalamic outputs driving these behavioral and metabolic responses. Here we provide evidence that central delivery of poly I:C is associated with activation of inflammatory signaling in the MBH, and activation of pathways regulating appetite and metabolic rate. Collectively, these data define a novel hypothalamic signaling pathway that is responsive to a common infectious PAMP, providing an avenue for CNS directed therapies that do not produce systemic immunocompromise.

## Results

### TLR3, TRIF, and IPS-1 signaling is not required for anorexia and body weight loss in response to poly I:C via central administration *in vivo*

**Figure 1:**
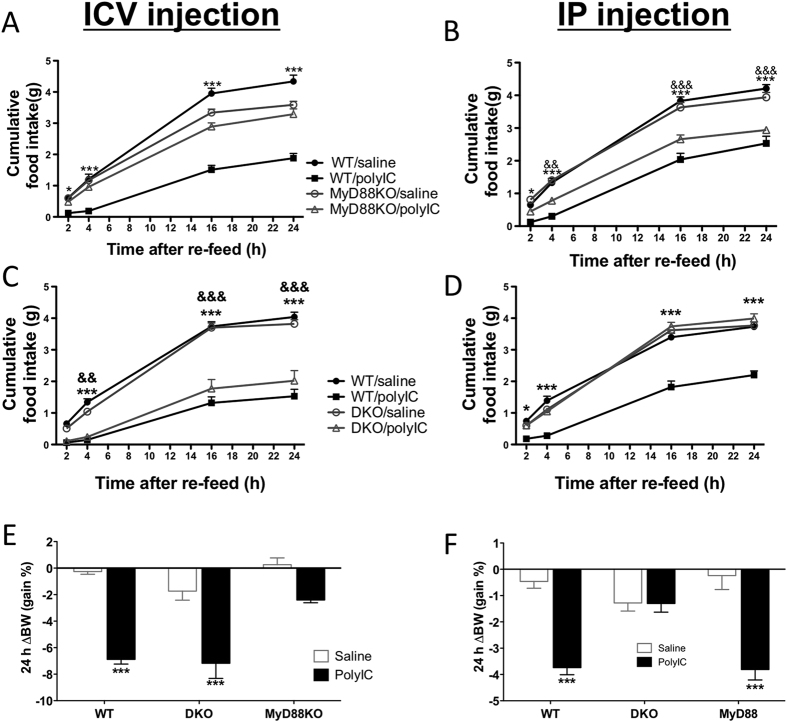
Central but not peripheral poly I:C administration- induced anorexia is mediated by MyD88. (**A,C**) Cumulative food intake (grams) during 24 h in WT versus MyD88KO mice (**A**) or WT versus DKO mice (**C**) following treatment with poly I:C versus saline via icv injection. (**B,D**) Cumulative food intake (g) during 24 h in WT versus MyD88KO mice (**B**) or WT versus DKO mice (**D**) following treatment with poly I:C versus saline via ip injection. Weight change over 24 hours in WT, DKO, and MyD88KO mice after poly I:C or saline given icv (**E**) or ip (**F**). Two-way ANOVA, WT/saline vs. WT/poly IC, *p < 0.05, **p < 0.01, and ***p < 0.001. KO/saline vs. KO/poly IC, ^&^p < 0.05, ^&&^p < 0.01, and ^&&&^p < 0.001. Error bars represent SEM. (See also Figs S1 and S2).

A number of studies, both *in vivo* and *in vitro*, demonstrate that TLR3/TRIF signaling is required for polyinosinic:polycytidylic acid (poly I:C)-induced inflammatory response, behavioral response (e.g. food intake, activity) and physical signs (e.g. fever, weight loss), that will hereafter be collectively called the sickness response^22^,^23^,^24^. However, prior studies focused on the response to peripheral injections *in vivo*, and on the response of circulating immune cells *in vitro*. We performed studies to determine whether TLR3/TRIF signaling is also essential in the CNS to produce the sickness response to poly I:C. We first performed nocturnal feeding experiments in TLR3KO and TRIFKO mice. These models do not directly address the issue of developmental compensation when either of these genes is deleted in the germline, so we generated TRIF/IPS-1 double knockout (DKO) mice as well. As expected, WT mice showed a significant decrease in food intake at all measured time points during the dark phase and marked body weight loss 24 h after receiving either icv or ip injections of poly I:C (Figs 1 and S1). In contrast, TLR3KO, TRIFKO, and DKO mice did not decrease food intake or lose body weight after receiving poly I:C via ip injection (Fig. 1D,F; Figs S1b and S2b). This is consistent with prior studies in TLR3KO mice, but to our knowledge is the first study of this feeding behavior in TRIFKO mice^25^. In contrast, TLR3KO, TRIFKO, and DKO mice responded to icv injection of poly I:C to the same degree as the WT cohort (Fig. 1C,E; Figs S1a and S2a). These observations indicate that TLR3/TRIF signaling is essential for the sickness response to peripheral poly I:C but is not required when poly I:C directly challenges the CNS. These experiments were repeated in female MyD88KO and WT mice with identical results (Fig. S2, Tables S1 and S2).

### RLRs/IPS-1 signaling is not necessary for anorexia and body weight loss in response to poly I:C following either central or peripheral administration

Signaling via the rig like receptors (RLRs; e.g. Rig-1, MDA-5) pathway is also important for the immune response to viral dsRNA, and in particular MDA5 senses longer viral dsRNA including the mimetic poly I:C^26^. To examine whether RLRs/IPS-1 signaling is necessary for the sickness response to poly I:C administered either directly into the CNS or into the periphery, we performed nocturnal feeding experiments in MDA5 KO and IPS-1 KO mice after either icv or ip injection. Both MDA5 KO and IPS-1 KO mice responded normally to both icv and ip injection of poly I:C (Figs S1a,b and S2a,b). These data demonstrate that RLRs/IPS-1 signaling is not necessary for anorexia and body weight loss induced by either central or peripheral administration of poly I:C.

### MyD88 signaling is required for anorexia and body weight loss in response to poly I:C after central but not peripheral administration

MyD88 is a universal adapter for all TLRs except TLR3, which is thought to signal exclusively via TRIF. Upon TLR (or IL-1R) activation, MyD88 is recruited to a signaling complex that in turn activates several signaling pathways leading to inflammatory responses^27^. To explore whether MyD88 is essential in the sickness response to central administration of poly I:C, we investigated the response of MyD88KO mice to icv or ip injection of poly I:C. Remarkably, MyD88KO mice exhibited normal feeding and unchanged body weight after receiving icv injections of poly I:C (Fig. 1A,E), whereas anorexia and weight loss after ip injection was indistinguishable from that found in WT mice at both short (2h) and long (24h) time points (Fig. 1B,F). The response was similar in both male and female mice (Tables S1 and S2) and younger or older MyD88 KO mice (not shown). Thus, MyD88 plays a key role in the response to poly I:C in the CNS, demonstrating that the signaling pathway essential for the sickness response to central poly I:C is distinct from that found in the periphery.

### MyD88 signaling is essential for fever and lethargy in response to poly I:C after central but not peripheral administration

**Figure 2:**
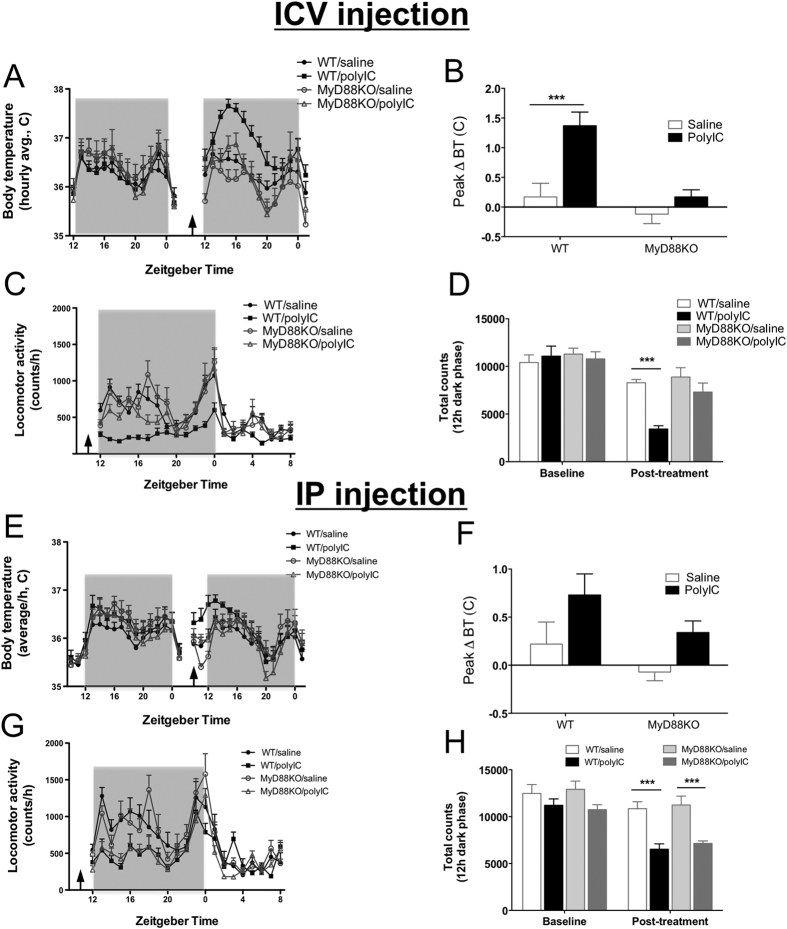
Central but not peripheral poly I:C administration-induced fever and inhibition of locomotor activity (LMA) requires MyD88 signaling. (**A**) Body temperature in WT versus MyD88KO mice before and after (arrow) receiving poly I:C versus saline via icv injection. BT data is expressed as hourly average. (**B**) Peak rise (Δ BT) in body temperature during 24 h in WT versus MyD88KO mice following poly I:C versus saline via icv injection. (**C**) Effect of icv poly I:C versus saline administration on voluntary LMA in WT versus MyD88KO mice. Movement data expressed as hourly sums. (**D**) Total voluntary LMA during 12-h dark phase in WT versus MyD88KO mice treated with poly I:C or saline via icv injection. (**E**) Body temperature in WT versus MyD88KO mice before and after (arrow) receiving ip poly I:C versus saline. BT expressed as hourly average (**F**) Peak rise in body temperature (Δ BT) during 24 h in WT versus MyD88KO mice following poly I:C or saline via ip injection. (**G**) Effect of ip poly I:C versus saline administration on voluntary LMA in WT versus MyD88KO mice. LMA expressed as hourly sums. (**H**) Sum total of voluntary LMA during 12h-dark phase in WT versus MyD88KO mice treated with poly I:C or saline via ip injection at ZT 9. Shaded regions indicates dark phase. ANOVA with post hoc testing, ***p < 0.001. Error bars represent SEM. (See also Fig. S3).

In animal models, poly I:C induces fever and lethargy in both mice and rats, and decreases both home cage locomotor activity (LMA) and voluntary wheel-running activity^28^,^29^. To examine whether poly I:C induces fever and lethargy when MyD88 is absent, we measured body temperature (BT) and LMA in MyD88KO mice following icv or ip injection of poly I:C. Compared to WT mice, MyD88KO mice exhibited neither fever nor diminished LMA after icv injection of poly I:C (Fig. 2A–D). However, similar to WT mice, there was significant reduction in LMA and an attenuated but significant rise in body temperature observed in MyD88KO mice after ip injection of poly I:C (Fig. 2E–H). In contrast, DKO mice demonstrated decreased LMA and fever only after central injection, but were resistant to ip injection of poly I:C (Fig. S3). These data demonstrate that MyD88 is essential for induction of fever and lethargy after central (but not peripheral) exposure to poly I:C, whereas TRIF/IPS-1 signaling is essential for responses in the periphery.

### TLR2, TLR4, TLR9 and IL-1R are not involved in poly I:C-induced anorexia and body weight loss

Although TLR3, and to a lesser extent the RLRs, are the primary receptors for dsRNA, the specificity of other TLRs for their classical ligands is poorly described in the CNS relative to the peripheral immune system. TLR4 is the receptor for lipopolysaccharide (LPS), a major immunostimulatory component of the cell wall of Gram-negative bacteria, and is the only TLR thought to signal through both TRIF- and MyD88-dependent pathways^6^. In peripheral immune cells, all other TLRs (except TLR3) require MyD88 as a part of their signaling complex. TLR9 recognizes viral DNA, TLR7 recognizes single strand RNA, and TLR2 forms homo- and heterodimers that recognize a wider array of PAMPs^26^. Similarly, IL-1R also requires MyD88 to elicit inflammation and associated sickness responses including fever, anorexia, lethargy and muscle catabolism^30^. To rule out the possibility of involvement of TLR2, TLR4, TLR7 and TLR9 in recognition of dsRNA, and to validate whether IL-1R is involved in amplifying the initial response to dsRNA, we performed feeding experiments with icv and ip injection of poly I:C in TLR2KO, TLR4KO, TLR7KO, TLR9KO and IL-1RKO mice. Similar to WT mice, all of these KO mice responded normally to poly I:C with both icv and ip injection (Fig. S2, Tables S1 and S2).

### MyD88 signaling plays a key role in up-regulation of brain inflammatory genes in response to poly I:C

**Figure 3:**
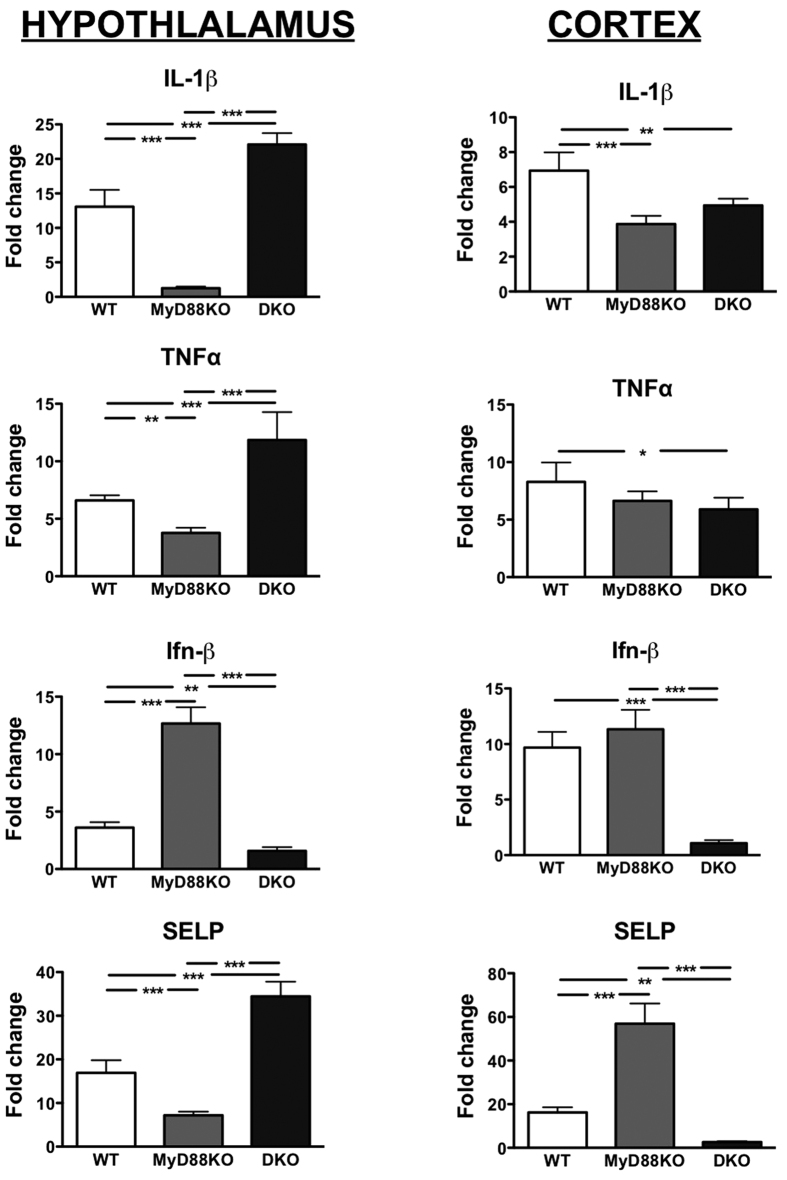
Induction of proinflammatory gene expression in hypothalamus and cortex after central poly I:C administration. Induction of IL-1β, TNFα, IFNβ, and SELP in mouse hypothalamus (left column) and cortex (right column). 6 h-post icv injection of saline or poly I:C, brains were extensively flushed by transcardial perfusion with PBS before dissection of hypothalami and cortices. Fold change indicates relative quantification (RQ) of mRNA in poly I:C-treated group vs. saline-treated group within each genotype. *p < 0.05, **p < 0.01, and ***p < 0.001. Error bars represent SEM.

Prior studies demonstrate that in response to both peripheral and central immune challenge, a pro-inflammatory response is generated within the CNS, with particularly robust inflammation noted within the MBH^15^,^16^,^18^,^31^. In particular, the local expression and release of IL-1β within the hypothalamus is critical for the induction of sickness behaviors^1^,^17^. Here, we confirm that central administration of Poly I:C leads to marked upregulation of IL-1β gene expression in the hypothalamus 6 hours after injection via a MyD88-dependent, but TRIF/IPS-1 independent signaling pathway (Two-way ANOVA treatment and genotype p < 0.0001; Treatment effect in WT and DKO both p < 0.001, MyD88KO n.s. by post hoc Bonferroni, (Fig. 3). A similar pattern was found for tumor necrosis factor alpha (TNFα, Fig. 3), C-C motif ligand 3 (CCL3, Fig. S4), and *SELP* (coding for the leukocyte adhesion molecule, p-selectin, Fig. 3). For C-C motif ligand-2 (CCL2), the induction of expression by Poly I:C was attenuated relative to WT, but remained significant (p < 0.001) in MyD88KO mice (Fig. S4). Expression of IL-6 was attenuated in both KO models relative to WT at this time point (Fig. S4). This does not represent global hypothalamic immune dysfunction in the MyD88KO model, as induction of specific anti-viral Ifnβ gene expression is more robust in the MyD88KO than in WT animals, but is absent in the DKO model (Fig. 3). A similar pattern was observed for CCL5, although the induction of this gene by Poly I:C remained significant for all genotypes (Fig. S4).

We also observed regional specificity to this response. The induction of IL−1β by Poly I:C in the cortex (Fig. 3) and cerebellum (not shown) was globally less robust than in the hypothalamus, and remained highly significant in the MyD88KO mouse (Poly I:C vs. Saline p < 0.01 for all genotypes, Fig. 3). In general, the induction of genes in the cortex showed a much stronger dependence on signaling via the classical pathway (i.e. was attenuated in the DKO model) and in the case of *SELP* the pattern of expression in the cortex was completely reversed relative to that found in the hypothalamus (Figs 3 and S4).

### MyD88 signaling plays an important role in hypothalamic leukocyte infiltration after chronic poly I:C challenge

**Figure 4:**
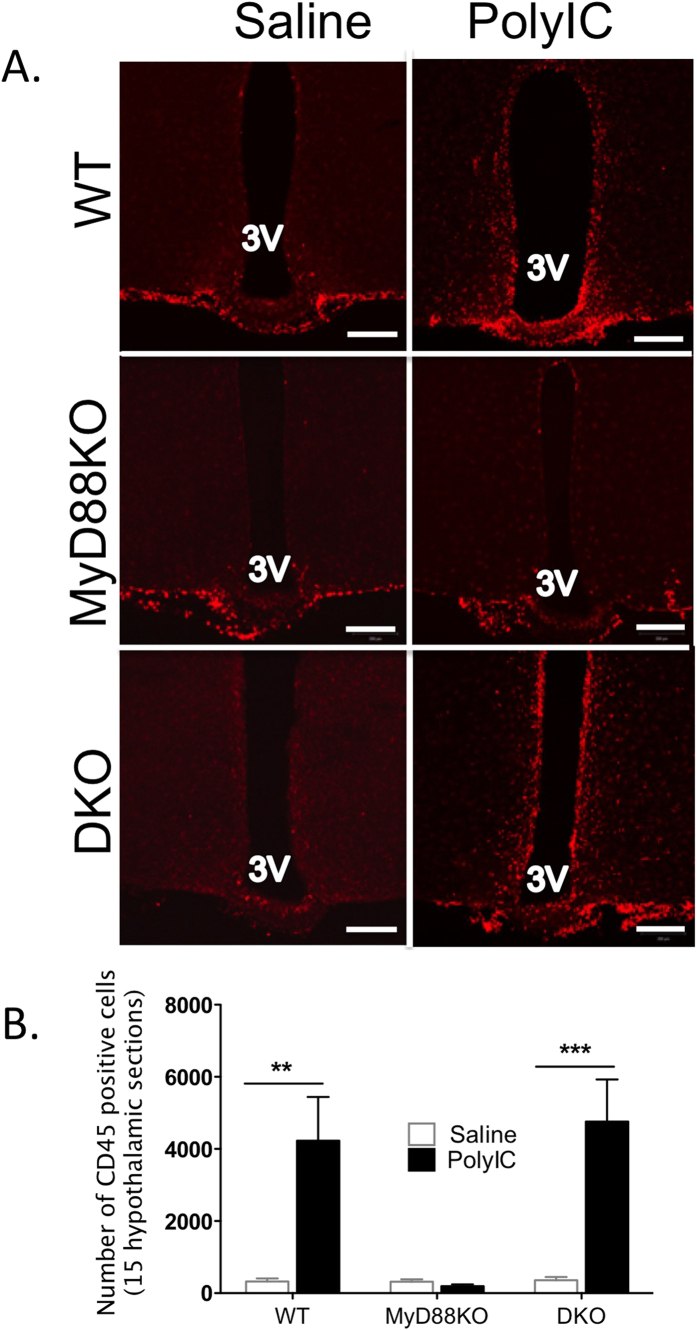
Chronic Poly I:C challenge robustly increases CD45 positive cells in WT and DKO but not MyD88DKO mouse hypothalamus. WT, MyD88KO and DKO mice were challenged via icv injection with two doses of poly I:C (total 4 μg/mouse) within 24 h. Brains were dissected after flushing and fixation. IHC was performed in brain sections using CD45 antibody. (**A**) Representative images of red-fluorescent CD45 positive cells in the MBH. (**B**) Total number of CD45 positive cells in 15 hypothalamic sections per mouse brain, spanning the rostral to caudal MBH were counted, and the average number of CD45 positive cells per hypothalamic section is shown. 3V = third ventricle. Two-way ANOVA, WT/saline vs. WT/polyIC, **p < 0.01. Error bars represent SEM.

Under normal physiological conditions, the hypothalamus contains few leukocytes. However, central inflammatory insults lead to rapid leukocyte recruitment into the CNS, a process that is required for prolonged illness responses in murine models of central viral infection^32^,^33^. We therefore analyzed the number of CD45 positive cells appearing in the hypothalamus after two central injections of Poly I:C (a total of 24 hours of exposure). Both WT and DKO mice had robust recruitment of round, CD45 positive cells into the MBH after poly I:C, whereas no increase in these cells was observed in the MyD88 KO mice (Fig. 4). DKO mice had nearly equivalent numbers of recruited cells as WT. We did not observe recruitment of these cells into the hypothalamus during the acute phase of illness (2 hours, not shown).

### Poly I:C activates the central melanocortin system, and activates brown adipose tissue via MyD88

**Figure 5:**
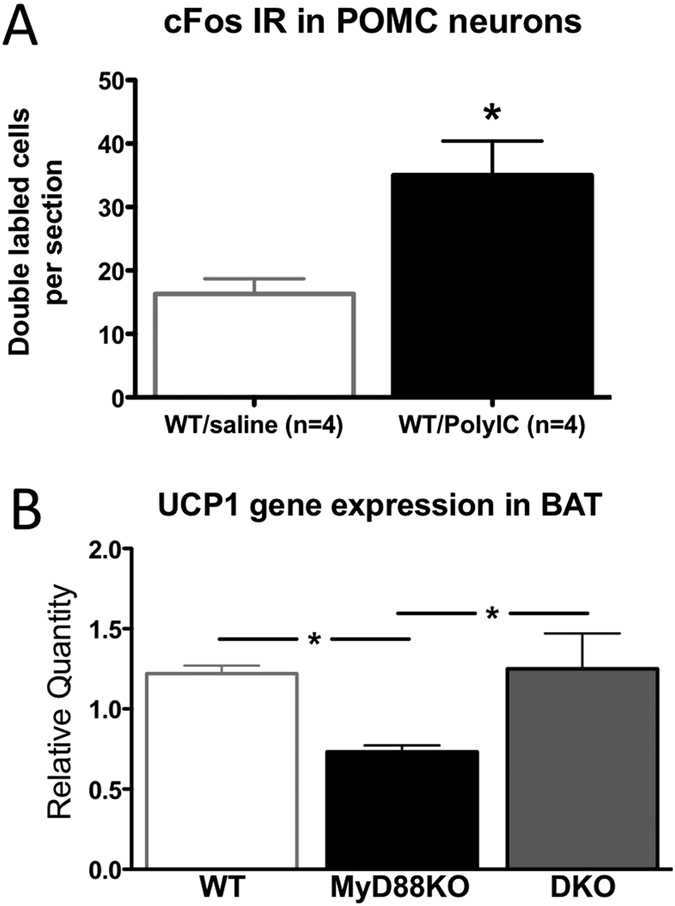
ICV Poly I:C challenge induces nuclear cFos in hypothalamic POMC neurons and increases UCP1 gene expression in BAT. (**A**) WT mice were challenged via icv injection with 20 μg of Poly I:C, then sacrificed and perfused 90 minutes later. Brains were processed and counted after blinding and randomization. Sections spanning the rostral-caudal distribution of POMC neurons were analyzed for number of POMC neurons with clear nuclear localization of cFos immunoreactivity. Average number of double-labeled cells/section is shown. *p < 0.05. (**B**) UCP1 gene expression in brown adipose tissue. 6 h-post icv injection of saline or poly I:C, brown adipose tissue (BAT) was dissected and RNA isolated. Fold change indicates mRNA relative quantification (RQ) in poly IC-treated group vs. saline-treated group within each genotype. Two-way ANOVA, post hoc *p < 0.01. Error bars represent SEM.

Previous studies demonstrated that anorexia and activation of sympathetic outflow by inflammatory insults is mediated at least in part by activation of central melanocortin signaling^17^,^34^,^35^. We therefore determined the level of cellular activation of hypothalamic POMC neurons utilizing nuclear localization of cFos protein as a general measure of cellular activation as previously described^17^. WT mice were injected with Poly I:C icv (20 mcg) or vehicle (n = 4 per group) and sacrificed 90 minutes later for blinded histochemical analysis. We found that there was a significant increase cFos nuclear localization in POMC neurons after central Poly I:C relative to saline treatment (Fig. 5A). In a separate study, groups of WT and MyD88KO animals were injected icv with Poly I:C and sacrificed 6 hours later. As an indirect measure of sympathetic outflow, we measured the expression of uncoupling protein-1 (UCP-1) in brown adipose tissue (BAT) as previously described^36^. There was a no difference in UCP-1 expression after saline injection, but we found a significant increase in UCP-1 gene expression in WT and DKO mice, but not in MyD88KO mice after Poly I:C injection (Fig. 5B). Collectively, these data argue that Poly I:C triggers a hypothalamic response similar to that found after exposure to other PAMPs (e.g. LPS), and activates sympathetic outflow via a central MyD88-dependent pathway.

### MyD88 signaling is critical for induction of inflammatory cytokine gene expression in microglia and astrocytes after poly I:C stimulation

**Figure 6:**
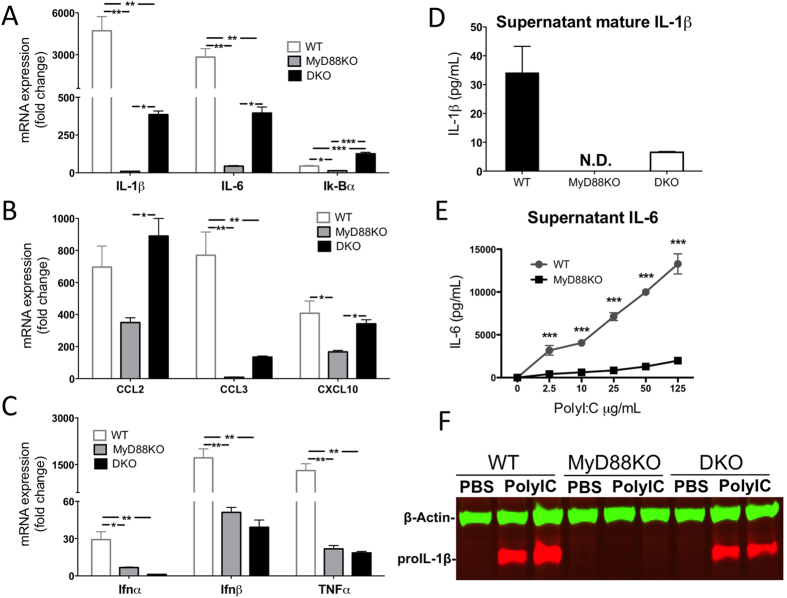
Attenuated inflammation in MyD88KO mixed-glial primary cultures relative to WT and DKO cultures. (**A–C**) Primary cultured mixed-glial cells from WT, MyD88KO and DKO mice, were stimulated with PBS or Poly I:C (50 μg/ml) for 6 h. Fold change indicates relative quantification (RQ) of mRNA in poly I:C-stimulated cells vs. PBS-stimulated cells. Error bars represent SEM. Similar results were observed in at least two independent experiments. (**D**) Mature IL-1β in supernatants measured by ELISA. Because IL-1β in MyD88KO cultures was not detectable (N.D.) no statistical analysis was performed. (**E**) IL-6 concentration in supernatants of WT and MyD88KO mixed-glial cells that were stimulated with PBS or 5 doses of poly I:C (2.5, 10, 25, 50 and 125 μg/ml). (**F**) Representative gel image from mixed-glial cells probed for pro IL-1β (red) and β-Actin control (green). *p < 0.05, **p < 0.01 and ***p < 0.001. Error bars represent SEM. (See also Figs S5 and S6).

Microglia are CNS resident innate immune cells, whereas astrocytes are the most numerous cell type in the CNS with potential to impact a wide range of homeostatic and pathological functions^37^. There are interactions and crosstalk between microglia and astrocytes during innate immune responses and inflammation^38^. We hypothesized that if the sickness behavior in response to central administration of poly I:C is directly mediated by MyD88-dependent activation of microglia and astrocytes, we would see a robust induction of inflammatory genes and a significant production of cytokines in mixed-glial cultures after poly I:C stimulation in WT cells but not in MyD88 KO cells. Primary mixed-glia cultures from WT, MyD88KO and DKO mice were stimulated for 6 h with PBS or with various doses of poly I:C, and then harvested and analyzed for expression of a variety of chemokines, cytokines and type I IFNs. We first confirmed that both TLR3 and TRIF expression was normal in unstimulated MyD88KO glia (data not shown). We then demonstrated that poly I:C induces robust and dose-dependent induction of a large number of inflammatory genes in WT mixed glial cultures, and that this response was greatly attenuated in MyD88KO cultures (Fig. S5). When compared at a fixed concentration of poly I:C (50 mcg/ml) we found significant attenuation of the expression of inflammatory cytokines (IL-1β, IL-6, TNF alpha; Fig. 6A,C), chemokines (CCL2, CCL3, CXCL10; Fig. 6B), and interferons (IFNα, Ifnβ; Fig. 6C) in MyD88 KO relative to WT mixed glial cultures. In contrast, glia obtained from DKO mice had robust upregulation of expression of IL−1β, IL-6, Iκ-Bα, CCL2, CCL3, and CXCL10 (Fig. 6A–C). The induction of IFNα, Ifnβ, and TNFα in DKO mice was similar to that found in MyD88KO mice, with both being attenuated relative to WT mice (Fig. 6A–C). As a positive control, we also tested the well documented interferon response to Poly I:C in peritoneal macrophages. As expected, both WT and MyD88KO macrophages had robust induction of IFNα and Ifnβ, whereas this response was attenuated in macrophages derived from TLR3KO and TRIFKO mice, and absent in DKO mice (Fig. S6a,b).

**Figure 7:**
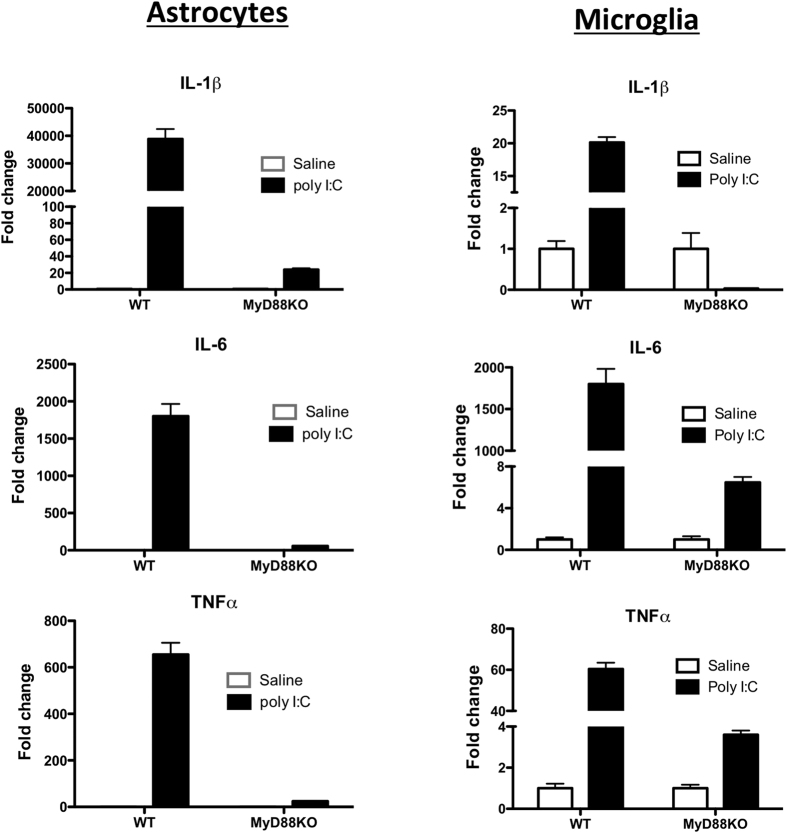
Poly I:C induced-gene expression of type I IFNs and cytokines was significantly diminished in MyD88KO astrocytes and microglia. Primary, purified cultures of astrocytes (left column) or microglia (right column) were obtained from WT and MyD88KO mice then stimulated with PBS or poly I:C (50 μg/ml) for 6 h. Fold change indicates relative quantification (RQ) of mRNA in poly I:C-stimulated cells vs. PBS-stimulated cells. Error bars represent SEM. Similar results were observed in at least two independent experiments. (See also Fig. S7).

We next sought to determine the relative role of each glial type in the response to poly I:C. Separate cultures of highly-enriched microglia or astrocytes were stimulated with poly I:C at 50 μg/ml for 6 h. Both microglia and astrocytes exhibit similar patterns of gene induction when activated by poly I:C (Results from two separate studies in Figs 7 and S6c,d), suggesting that each cell type contributes independently to the observed changes in mixed glia and *in vivo.* Because we analyzed each cell type individually relative to its own control, we did not directly address any potential synergism between these two cell types.

### MyD88 signaling plays critical role in synthesis and release of cytokines in microglia and astrocytes after poly I:C stimulation

Our gene expression analysis consistently demonstrated that the induction of mRNA expression for pro-inflammatory cytokines (IL-1β, IL-6, and TNFα) in the CNS, both *in vivo* and *in vitro*, required intact MyD88 signaling. In order to demonstrate an impact on protein translation of this transcriptional event, we analyzed the cellular content or release of cytokines by primary mixed glial cultures. We confirmed that the synthesis of pro- IL-1β in mixed glial cells was nearly undetectable at baseline, but expressed at high levels 6 hours after stimulation with Poly I:C in cells derived from both WT and DKO mice, but not those obtained from MyD88KO mice (Figs 6D and S7a) We also detected mature IL-1β production in the conditioned media, demonstrating activation of the inflammasome (Fig. 6D). Similarly, IL-6 was released into the supernatant at high levels in a dose-dependent fashion in all WT cultures, but release of this cytokine in MyD88KO-derived cultures was minimal (Fig. 6E). In contrast, and in agreement with our mRNA analysis, production of the chemokine CXCL10 was not dependent on either TRIF/IPS-1 or MyD88 in the mixed glial cultures, with cells derived from all genotypes releasing this chemokine to the same extent (Fig. S7b). Thus, transcription, translation, and release of pro-inflammatory cytokines by microglia after Poly I:C stimulation is dependent on MyD88 signaling, and is largely independent of signaling via previously described dsRNA response pathways requiring either TRIF or IPS-1. In contrast, the synthesis and release of at least some chemokines is only moderately affected by loss of MyD88 signaling, but is nonetheless also independent of signaling via TRIF or IPS-1. In some cases (e.g., interferons, TNFα), both MyD88 and either TRIF or IPS-1 contribute equally to the transcriptional response. Collectively, these data demonstrate that the inflammatory response to Poly I:C by central glia is complex, but dominated overall by an as yet undefined pathway that requires MyD88 in the signaling complex.

## Discussion

The data from our previous work and that from other groups demonstrates a unique role for hypothalamic MyD88 signaling in the illness response to several important PAMPs, and in the inflammatory amplification and cytokine signaling observed in the hypothalamus during systemic inflammation^17^,^31^. Furthermore, *in vitro* stimulation of TLR3KO microglia with poly (I:C) results in substantial cytokine release and activation of downstream MAPK signaling, albeit with delayed kinetics relative to WT microglia^24^. Our data demonstrate that induction of key pro-inflammatory cytokines (e.g. IL-1β, IL-6, TNFα) in response to poly I:C also occurs in the hypothalamus *in vivo*, and that MyD88 (but not TRIF or IPS-1) is the dominant transducer of this response. However, Town *et al*. demonstrated that morphological evidence of microglial activation (i.e. microgliosis) after icv injection of Poly(I:C) was TLR3 dependent, and our own data demonstrate that a subset of the chemokine response to Poly(I:C) occurs normally in MyD88KO microglia, demonstrating that parallel cellular pathways are activated by this stimulus^24^. We also show that numerous lymphocyte recruitment genes (e.g., selectins, chemokines) are upregulated in the hypothalamus in response to these same insults, many via a MyD88-dependent pathway. This is also an important finding, because central lymphocyte recruitment is requisite for sustaining wasting in chronic viral illness^33^,^39^,^40^.

In addition to endosomal TLR3 sensing, dsRNA is also sensed by RIG-1-like helicases (RLHs) in the cytosol. Members of this family include melanoma differentiation-associated gene 5 (MDA5) and retinoic acid-inducible gene 1 (RIG-1)^7^. While TLR3 signals exclusively through TRIF, both RLHs signal through a different adapter, IFNβ promoter stimulator 1 (IPS-1). The relative contributions of these dsRNA sensors to viral immunity, including central responses to virus, are unclear. Although RLHs are not known to link directly to MyD88-dependent pathways, we nonetheless investigated the possible role of RLH signaling in the link between peripheral and central poly (I:C) delivery and sickness behavior. We found no *in vivo* role for either MDA5 nor the common adapter IPS-1 in the anorexia or weight loss induced by poly (I:C), regardless of the route of administration. To address the issue of developmental compensation *in vivo*, and to provide an *in vitro* model that eliminated both TRIF and IPS-1 dependent signaling, we created a DKO model lacking both of these signaling proteins. These mice responded similarly to the TLR3KO in our *in vivo* testing, confirming that the sickness response to central poly I:C is both TRIF and IPS-1 independent.

An important limitation of our *in* vivo studies is that we delivered poly I:C directly into the ventricular system. However, there are numerous ways for both viruses and dsRNA to enter the brain from the periphery, all of which are documented for various viral infections. These include directly crossing the blood-brain barrier (BBB) or by circumventing the BBB via several different routes of entry. However, the most critical data relevant to our study comes from publications demonstrating that peripheral injections of poly I:C or peripheral inoculation of West Nile Virus lead to substantial breakdown of the BBB via a TLR3-dependent mechanism, providing ready access of dsRNA (or dsRNA generating viruses) to the brain^41^,^42^. We hypothesize that the behavioral response to peripheral poly I:C is in part determined by classic TLR3/TRIF pathway-induced attenuation of the BBB, which in turn facilitates viral PAMP signaling via our novel central MyD88-dependent pathway. While only three gross brain regions were examined (cortex, cerebellum vs. hypothalamus), another key finding is that of regional specificity. In the case of IL-1β, the induction in the hypothalamus was both more robust than in the cortex or cerebellum, and also more obviously dependent on MyD88 signaling. For the lymphocyte recruitment gene *SELP*, the pattern observed in the hypothalamus and cortex was reversed, and its induction was MyD88-dependent only in the hypothalamus. In the case of Ifnß, the induction of expression *in vivo* was entirely dependent on TRIF and/or IPS-1, suggesting that antiviral interferon signaling is initiated via the same pathways found in the peripheral immune system. Interestingly, interferon expression *in vitro* was attenuated to a similar degree in both MyD88KO and DKO primary cultures, suggesting that the result *in vivo* is in part due to redundant signaling in non-glial cells. Collectively, these data argue that MyD88 is critical for the initiation of the hypothalamic innate immune response to a key viral PAMP and that neither TRIF nor IPS-1 is absolutely required for this process. It is noteworthy that in the hypothalamus, the induction of pro-inflammatory mRNA transcripts in the DKO animals typically *exceeded* that of WT animals, suggesting that the TRIF or IPS-1 pathway may serve to limit or dampen the inflammatory response in this context.

Our data also demonstrates that MyD88 is essential for recruitment of lymphocytes to the hypothalamus but does not eliminate the possibility that this is due to deletion of MyD88 in the lymphocytes themselves. Indeed, Zhou *et al*. utilized MyD88KO mice and adoptive transfer of T cells to demonstrate that intact MyD88 signaling in CD4+ T cells was required to produce chronic wasting in a murine viral encephalitis model^43^. However, the early phase of illness and wasting (occurring after the first few days of intravenous infection) is independent of CD4+ T-cell function in this same model^44^. Furthermore, this infection produces a prompt and robust inflammatory response arising exclusively from the innate immune system in the brain, accompanied by transition of microglia from ‘resting’ to “activated” morphology^45^. We hypothesize that the innate immune system in the brain (including microglia) responds to the initial viral insult with the production of pro-inflammatory cytokines and chemokines, as well as with a transition to an activated, antigen-presenting state. The initial wave of sickness responses are supported by pro-inflammatory cytokines (e.g. IL-1β), with illness being sustained by central recruitment of adaptive immune cells that require normal MyD88 function for full effect. The lack of lymphocyte recruitment into the hypothalamus that we observed during the early stage of acute illness (two hours after Poly I:C) is consistent with this hypothesis.

Our *in vitro* analysis confirms the basic findings of the *in vivo* studies, and suggests several general principles. First, the expression patterns of the key hypothalamic pro-inflammatory cytokines (IL-1β, IL-6) were reflected by that found in the glial cultures, with the induction of their expression by Poly I:C being more dependent on MyD88 than on TRIF/IPS-1. For most genes studied, MyD88 deletion attenuated the poly I:C induced expression within the intact hypothalamus to a lesser extent than that observed in cultured glial cells, likely reflecting the presence of TLR3-TRIF dependent signaling in non-glial cells in the hypothalamus. Second, the expression patterns observed in isolated microglia was remarkably similar to that found in isolated astrocytes suggesting that these two cell types both utilize a MyD88 dependent signaling pathway for sensing poly I:C. Furthermore, both cell types showed normal MyD88-independent induction of a subset of genes by poly I:C (e.g. CCL2, CCL5, CXCL10), demonstrating that MyD88 deletion does not render these cells globally resistant to immune signaling and that parallel pathways exist in these cells for the production of the overall immune response. Finally, the mRNA expression of cytokines with the most potent behavioral impact in the brain, IL-1β and IL-6, are directly reflected by the production of the respective proteins. WT mixed glial cells demonstrated a strongly dose-dependent release of IL-6 in response to poly I:C, and this response was not observed in MyD88KO glia. Similarly, pro IL-1β protein synthesis (measured in cell extracts) and mature IL-1β release (measured in supernatants) was strongly induced in WT and DKO cells, but this response was absent in MyD88KO cultures. The cell type responsible for the production of mature IL-1β in our studies was not determined, but previous studies strongly suggest that microglia are the sole source of this cytokine under these conditions^46^. In sum, the *in vitro* data provide support for a model wherein the primary link between central exposure to this key viral PAMP and sickness behavior is MyD88-dependent and likely mediated by microglia and astrocytes. Because of the relatively small tissue volumes, we did not address the issue of regional specificity with our *in vitro* studies. However, the *in vivo* analysis does indicate that such regional specificity exists. Future studies will be necessary to determine the degree to which this reflects the complex circumventricular anatomy (with associated fenestrated blood vessels, tanycytes, etc.) of the MBH, or is instead an intrinsic property of the glia in these regions.

The observation that IL1-RKO mice respond normally to central Poly I:C suggests that this MyD88 dependence is not simply a reflection of the induction and action of IL-1β within the MBH. However, peripheral or central injection of IL-1β leads to neuronal activation in several brain regions, with the most obvious activation evident in nuclei that make up the MBH and associated vascular structures^17^,^47^. Both IL-1β and its receptor are highly expressed in this brain region during systemic inflammation, and central IL-1β potently induces sickness behaviors and wasting in murine models^15^,^18^. Thus, it is likely that this cytokine plays a role in the sickness response to poly I:C in WT animals, with other redundant signals (e.g. IL-6) playing a dominant role with lifetime whole body IL-1R deletion.

There is also some evidence that poly I:C activates cells in the MBH, although this has not been studied in detail^48^. MBH neurons expressing POMC and adjacent neurons expressing agouti-related peptide (AgRP) provide powerful anorexigenic and orexigenic drive, respectively. POMC neurons release the anorexic neurotransmitter αMSH that binds to type 4 melanocortin receptors (MC4R) in a number of downstream sites in the brain. AgRP functions as an antagonist of this receptor and AgRP neurons directly inhibit POMC neuronal activity, producing increased appetite and energy storage [for a recent review, see ref. 49]. Our work and that of others demonstrated that POMC and AgRP neurons are central targets of cytokine signaling and provide an important primary neuronal substrate linking inflammation to anorexia and catabolism^17^,^50^,^51^. Here, we demonstrate that hypothalamic POMC neurons are activated by this PAMP, thereby providing a neuronal substrate for a subset of behavioral and metabolic sickness responses in this model. Further, we demonstrate increased expression of UCP-1 in BAT in response to this same stimulus, strongly suggesting activation of sympathetic outflow and increased thermogenesis^52^. This metabolic response was present in WT animals but not in MyD88KO animals and is consistent with the attenuated febrile response in this model. This demonstrates that MyD88 is critical for at least a subset of metabolic sickness responses in addition to behavioral alterations with CNS exposure to poly I:C.

Our studies support the general idea that the core of the illness response to viral infection lies at the intersection of the CNS immune system and brain centers regulating appetite, metabolism, arousal, and nutrient partitioning. Further, our data demonstrate that sickness behaviors and metabolic responses to central poly I:C require intact MyD88 signaling. Most importantly, the molecular pathway leading from poly I:C stimulation to cellular activation of CNS immune cells is distinct from that found in the periphery, thereby creating a pathway for therapeutic intervention during the evolution and maintenance of viral illness that would alleviate debilitating symptoms without ameliorating the global inflammatory response needed to fight viral infection.

## Methods

### Mice

Knockout mice of TLR2, TLR3, TLR4, MDA5, IL-1R, MyD88, TRIF, IPS-1 and age-sex-matched C57BL/6J wild type (WT) mice were purchased from The Jackson Laboratory (Bar Harbor, ME). TLR9 KO mice were provided by Dr. Mary Stenzel-Poore. Double knockout (DKO) mice of TRIF/IPS-1 were generated by intercrossing TRIFKO and IPS-1KO mice as described previously^53^,^54^. KO and DKO mice were genotyped using standard protocols from The Jackson Laboratory. All mice were housed in rooms with controlled temperature (25 ± 2 °C) and illumination (12 h light/12 h dark) and provided *ad libitum* access to water and food (Purina rodent diet 5001; Purina Mills, St. Louis, MO, USA) unless otherwise stated, and were allowed to acclimate for at least 7 d before procedures. Adult mice between 8–12 wk of age were used for *in vivo* experiments, and newborn mice (postnatal day 1 to 3) generated from our own breeding colonies were used for *in vitro* experiments. All studies were conducted according to the National Institutes of Health Guide for the Care and Use of Laboratory Animals and approved by the Institutional Animal Care and Use Committee of Oregon Health & Science University.

For *in vivo* experiments, one day before each experiment commenced, the animals were weighed and divided into treatment groups such that the mean body weights of each group were similar. During the experiments, food intake and body weight were measured at the same time each day, unless otherwise noted.

### Intracerebroventricular and Intraperitoneal Injections

Under isofluorane anesthesia, 26-gauge lateral ventricle cannulas (PlasticsOne, Roanoke, VA, USA) were placed in mice using a stereotactic alignment instrument (Kopf, Tujunga, CA, USA) at the following coordinates relative to bregma: 1.0 mm X, −0.5 mm Y and −2.25 mm Z. Mice were then individually housed and allowed to recover from surgery for at least 7 days. Poly I:C (Sigma, St. Louis, MO, USA) was dissolved in 0.9% saline according to the manufacturer’s instruction. 0.9% saline or 20 μg of poly I:C (per mouse) was given in 2 μl total volume through intracerebroventricular (icv) injection. For peripheral treatment, 0.9% saline or poly I:C at 10 mg/kg body weight was injected intraperitoneally (ip).

### Body Temperature and Locomotor Activity Measurement

Body temperature (BT) and voluntary home cage locomotor activity (LMA) were measured using a MiniMitter system (MiniMitter, Bend, OR, USA) as described previously^31^,^55^. Briefly, under isofluorane anesthesia, transponders for sensing BT and LMA were implanted adjacent to the abdominal aorta in the retroperitoneal space. Mice were allowed to acclimate for at least 7 days before BT and movement in x-, y- and z-axes were recorded in 5- minute intervals (Vital View, MiniMitter). Δ BT was calculated in B and D using formula Δ BT = BT_post-injection_ − BT_pre-injection_ at a same ZT point during the 48 h period of before and after treatment.

### Nocturnal Feeding Studies

All feeding studies were performed at night, as described previously^36^. Mice were individually housed for conditioning for at least 7 days. 2 days before treatment, mice were placed in clean individual cages with measured amounts of food. Baseline body weight change and 24 h food intake were obtained on two consecutive days. On the experimental day, 3h before lights off, mice were administered poly I:C at 20 μg/mouse (icv) or 10 mg/kg body weight (ip), respectively, and pre-weighed food pellets were placed into each cage at 5:00 PM. Food was weighed at 4 time points (2 h, 4 h, 16 h and 24 h). Body weights were measured at 2 time points (16 h and 24 h). Care was taken to minimize stress and light exposure to the animals during the nighttime of food measurements.

### Tissue Collection

For RT-PCR analysis in tissues, 6 h after icv injection, mice were deeply anesthetized using a ketamine cocktail and sacrificed by trans-cardiac perfusion (≥50 ml) with PBS to remove intravascular blood. Brains and inter-scapular brown adipose tissue (BAT) were immediately removed. Hypothalamic and cortical blocks were dissected. Hypothalami, cortices and BAT were snap frozen and stored in −80 °C until analysis. For immunohistochemistry (IHC) analysis of lymphocytes in brain tissue, after two icv injections (two doses/mouse within 24 h), mice were perfused by PBS followed by 4% paraformaldehyde (PFA) for tissue fixation. Brains were post-fixed in 4% PFA for overnight and cryoprotected in 20% sucrose for 24 h at 4 °C before being stored at −80 °C until IHC analysis.

### Primary Culture

#### Mixed-Cultures

Primary mixed-cultures of microglia and astrocytes were prepared from neonatal mouse cortices as described previously^56^ with modifications. Briefly, under sterile conditions cerebral brains from newborn mice were dissected, freed of the meninges, and were kept on ice before digestion using papain (Worthington, Biochemical Corporation). Dissociated mixed-cells in complete medium (DMEM low glucose with L-glutamine, 10% FBS and 1% penicillin/streptomycin) were seeded in 75-cm^2^ flasks or 100-mm dishes. Primary mixed-cultures were incubated for 14–16 days by feeding complete medium twice a week so that only glial cells remained. For RT-PCR in mixed-cultures, cells were harvested and re-plated into 6-well plates (Falcon; BD Bioscience) at 1 × 10^6^ cells/well with complete medium for 1–2 days before stimulation. For Western blots in mixed-cultures, cells were cultured in 100-mm dishes for 14–16 days until stimulation.

#### Highly-Enriched Microglia

As previously described^38^,^56^, highly-enriched microglia were isolated from mixed-cultures by shaking flasks at 200 rpm at 37 °C for 2 h in an incubator-shaker. Cells were re-plated into 6-well plates at 5 × 10^5^/well and maintained with complete medium for overnight before stimulation. More than 99% of these isolated cells were confirmed as microglia by ICC Iba1 staining.

#### Highly-Enriched Astrocytes

We utilized cultures containing L-leucine methyl ester (LME, Sigma, St. Louis, MO, USA) in mixed-cultures^38^,^57^. Briefly, mixed-cells were harvested from newborn mouse cortices and seeded into 6-well plates at 1.2–1.5 × 10^6^ cells/well with complete medium described above. 72 h after seeding, LME at 1 nM (final concentration) was applied in complete medium for 6 days before stimulation (medium with LME was changed once during 6-day culture period). More than 99% of these cells stained positive for GFAP by ICC, confirming their identity as astrocytes.

#### Peritoneal Macrophages

Mouse peritoneal macrophages were harvested after elicitation with 3% Brewer thioglycollate medium for 4 days as described previously^58^. Cells were re-plated into 6-well plates at 5–10 × 10^6^ cells/well and maintained with DMEM/F12-10 medium overnight before stimulation.

### Cell Stimulation and Sample Collection

For RT-PCR in mixed-cultures, cells were stimulated with PBS or poly I:C at 50 μg/ml for 6 h. For RT-PCR or for Western blots, cells were stimulated with PBS or poly I:C at 50 μg/ml for 6 h. During sample collection, culture supernatants were first collected, and adherent cells were washed and lysed using cell lysis buffer (RT-PCR, RLT buffer, Qiagen Inc. Valencia, CA; Western blots, Cell Signaling, Danvers, MA, USA). RNA preparation and protein extraction were performed as described previously^31^,^36^. RNA and protein samples were stored in −80 °C until analysis.

### Quantitative RT-PCR

Real-time quantitative PCR (RT-PCR) was performed as described previously^36^. RNA was extracted from tissues or cells using RNeasy kits (Qiagen Inc., Valencia, CA). Reverse transcription and quantitative PCR reagents were obtained from Life Technologies (Carlsbad, CA). 18S, β-actin or GAPDH cDNA were used as endogenous control. Gene expression is reported as mRNA fold change relative to saline or PBS treated group within genotype of mice or cells using the 2^−ΔΔCt^ method. Statistical analyses were performed on the ΔCt values.

### Western Blot

Protein synthesis of inflammatory cytokines in mixed-cultures was measured by Western blots. 35 μg per lane (IL-1β) or 5 μg per lane (CXCL10) of total protein extracted from mixed-cultures was run on Novex 10–20% Tris-Glycine gels (Life Technologies) at 120 V. Gels were transferred to Immobilon-FL membranes (Millipore) and blocked with 5% BSA for 1 h. Membranes were incubated o/n with β-actin mouse mAB 1:4000 (Cell Signaling, #3700) and either IL-1β Rabbit mAB 1:1000 (Cell Signaling, #12426) or Anti-IP10 (CXCL10) Rabbit 1:1000 (Abcam, #ab9938). After 4x washing with TBST, membranes were incubated for 1 h with DyLight 680 Anti-rabbit and 800 Anti-mouse 1:15000 ea (Cell Signaling), then visualized.

### ELISA

IL-6 or IL-1β in culture supernatants were measured using ELISA kits according to manufacturing instructions (IL-6 kit was purchased from BD Biosciences; IL-1β kit was purchased from eBioscience).

### Immunohistochemistry

Primary cultured mixed glial cells and highly-enriched microglia and astrocytes were cultured directly on poly-D-lysine/laminin coated glass coverslips (Corning Biocoat, German) in 24-well plates at 1–2 × 10^5^ cells/ml with complete medium for 24 h. Cells were washed with PBS and fixed with 4% paraformaldehyde for 20 min on ice. Using a similar protocol as described previously^36^. For brain histochemistry, mice were sacrificed and brains processed as described previously^17^.

#### Cultured glia

Immunofluorescent staining was processed with primary antibodies GFAP diluted 1:1000 (MAb360, Millipore) and Iba1 diluted 1:500 (Wako) and secondary fluorescent antibodies (goat anti-mouse Alexa Flour 594 with 1:500 dilution or goat anti-rabbit 488 with 1:500 dilution; Invitrogen) for visualization. In the negative controls, primary antibodies were omitted. Cell nuclei were labeled with DAPI at 1: 25,000 dilution for 5 min.

#### Hypothalamic CD45 Cells

IHC in brain tissue was performed as described previously^36^. Briefly, four series of 30-μm coronal sections were cut and collected throughout the hypothalamus. One series of hypothalamic sections was processed for CD45 IHC. After washing and blocking, floating sections were incubated with CD45 primary antibody (rat anti-mouse CD45, 1:1000; BD Biosciences) for overnight at 4 °C, followed by incubation with secondary antibody conjugated with Alexa Flour 555 (1:500; Invitrogen) for visualization. Sections were viewed and the number of CD45 immuno-fluorescence positive cells was manually counted under a fluorescent microscope (model 4000 DM, Leica Microsystems).

#### POMC neurons

For POMC neuronal staining, we used anti-POMC (rabbit antibody, 1:5000 dilution, Phoenix Pharmaceuticals) and anti-cFos (goat antibody, 1:25000 dilution, Santa Cruz). Secondary antibodies were donkey anti- rabbit (Alexa Flour 594, 1:500, red for POMC) and donkey anti- goat (Alexa Flour 488, 1:500, green for cFos, Invitrogen). Rostral-caudal distribution of sections for each hypothalamus was matched prior to blinded cell counting. Confocal photomicrographs were taken using a Zeiss LSM700 confocal microscope (Carl Zeiss, Oberkochen, Germany) under identical microscope settings. Images were processed using NIH ImageJ software.

### Statistical Analysis

All data are expressed as mean ± standard error of the mean (SEM) for each group. Statistical analyses were performed using the unpaired Student’s t-Test or ANOVA followed by Bonferroni posttests using GraphPad Prism 5 (La Jolla, CA). p < 0.05 was considered statistically significant.

## Additional Information

**How to cite this article**: Zhu, X. *et al*. A distinct brain pathway links viral RNA exposure to sickness behavior. *Sci. Rep.* **6**, 29885; doi: 10.1038/srep29885 (2016).

## Supplementary Material

Supplementary Information
