# Supplementary material for: A distinct brain pathway links viral RNA exposure to sickness behavior
Source: Sci Rep. 2016 Jul 20;6:29885. doi: 10.1038/srep29885 (PMC4951726; doi:10.1038/srep29885)
Supplement: Supplementary Information [file srep29885-s1.pdf]

DATA SUPPLEMENT:

A distinct brain pathway links viral RNA exposure to sickness behavior.

Xinxia Zhu,[1] Pete R. Levasseur,[1] Katherine A. Michaelis,[1,2] Kevin G. Burfeind,[1,2] and Daniel L. Marks[1,*]

[1] Papé Family Pediatric Research Institute, Oregon Health & Science University, Portland, OR 97239, USA

[2] MD/PhD Program, Oregon Health & Science University, Portland, OR 97239, USA

*Correspondence: marksd@ohsu.edu

Figure S1.

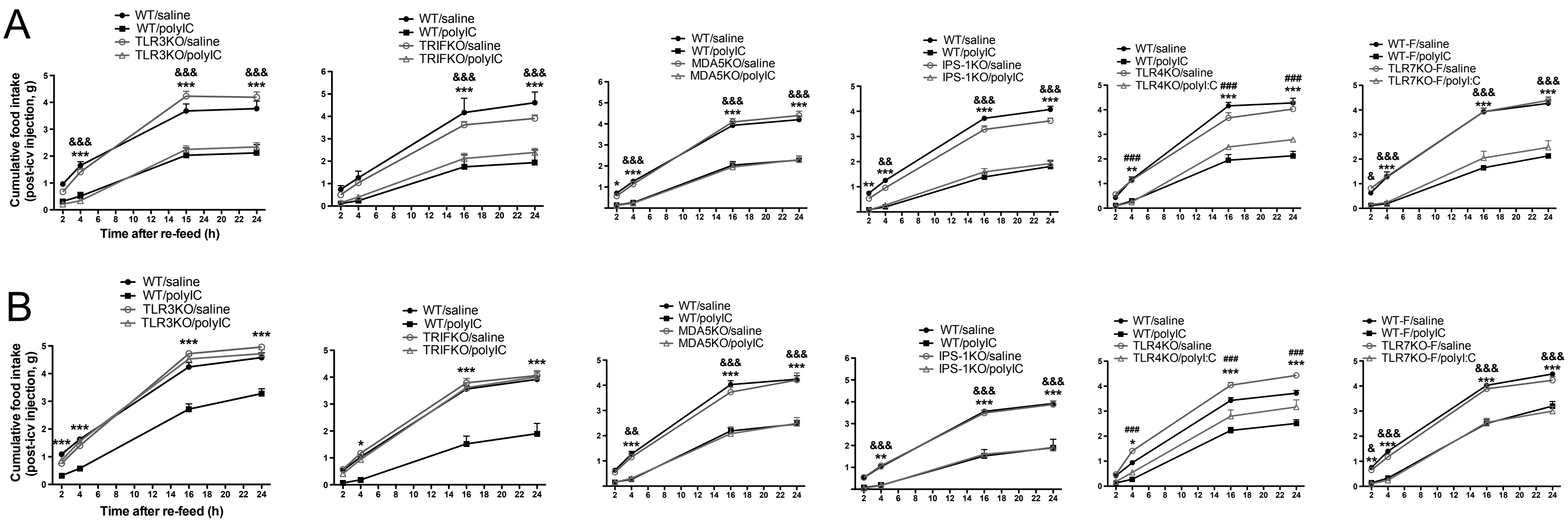

Figure S1, related to Figure 1. Central but not peripheral poly I:C administration- induced anorexia is mediated by MyD88.

(A) Cumulative food intake (grams) during 24 h in WT versus TLR3KO, TRIFKO, MDA5KO, IPS-1KO, TLR4KO, and TLR7KO mice following treatment with poly I:C versus saline via **icv** injection.

(B) Cumulative food intake (g) during 24 h in WT versus TLR3KO, TRIFKO, MDA5KO, IPS-1KO, TLR4KO, and TLR7KO mice following treatment with poly I:C versus saline via **ip** injection.

Two-way ANOVA, WT/saline vs. WT/poly IC, *$p < 0.05$, **$p < 0.01$, and ***$p < 0.001$. KO/saline vs. KO/poly IC, &$p < 0.05$, &&$p < 0.01$, and &&&$p < 0.001$. Error bars represent SEM.

Figure s2.

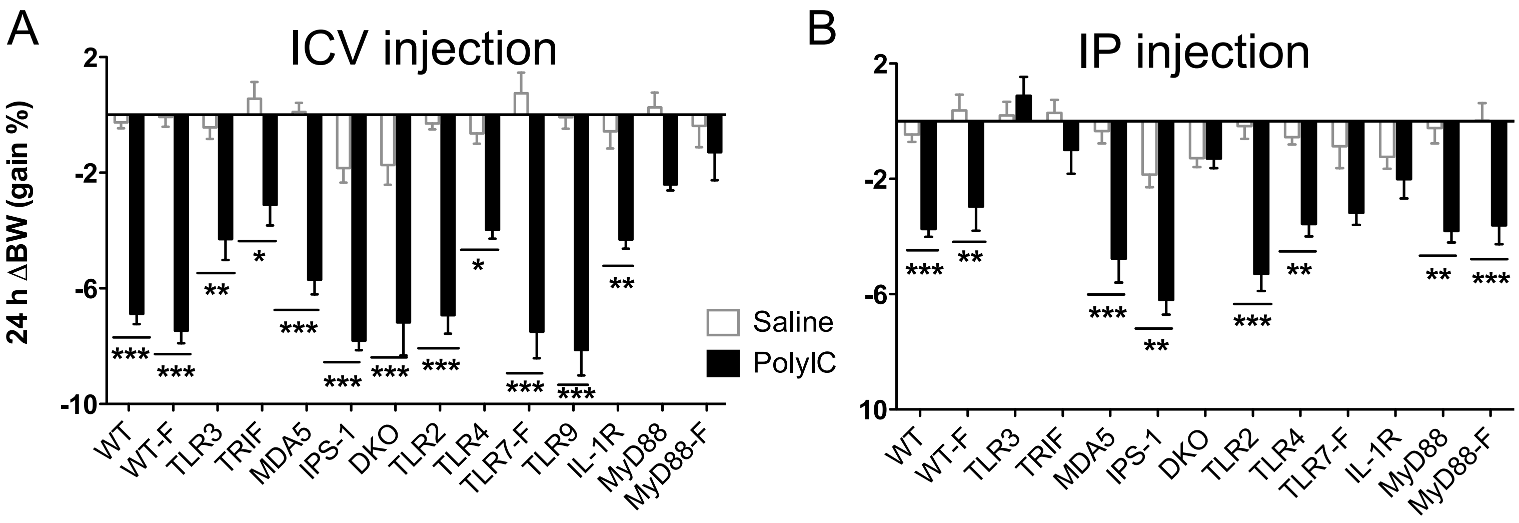

Figure S2, related to Figure 1. Central but not peripheral poly I:C administration- induced body weight loss is mediated by MyD88.
(A) Body weight change during 24h in WT versus MyD88KO and other types of KO mice following treatment with either poly I:C or saline via **icv** injection.
(B) Body weight change during 24 h in WT versus MyD88KO and other types of KO mice following treatment with either poly I:C or saline via **ip** injection. Body weight change (Δ BW) expressed as % of baseline body weight.
Two-way ANOVA, *p < 0.05, **p < 0.01, and ***p < 0.001. Error bars represent SEM.

Figure S3.

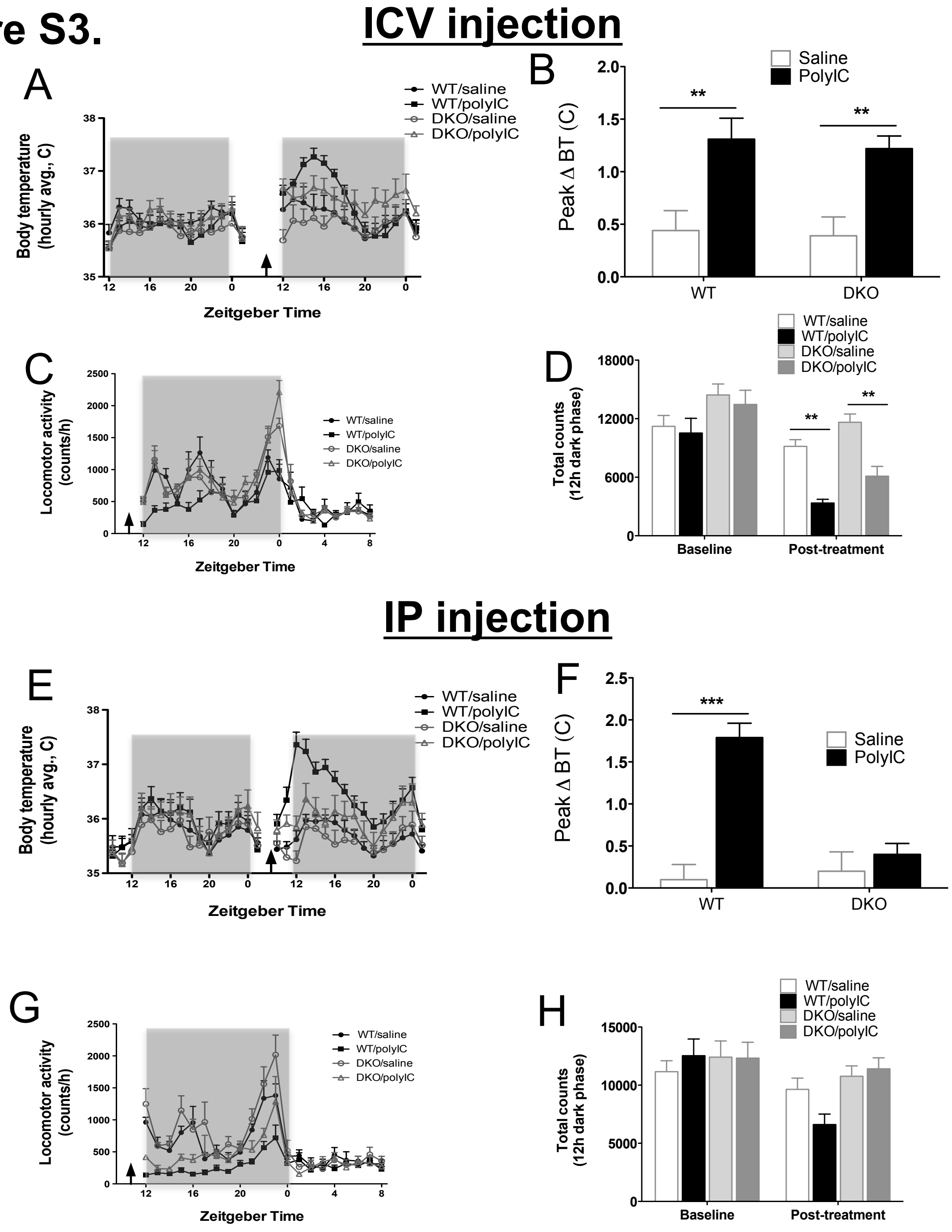

Figure S3, related to figure 2. Central but not peripheral poly I:C administration-induced fever and inhibition of locomotor activity (LMA) is not dependent on TRIF/ IPS-1 signaling.
(A) Body temperature in WT versus DKO mice before and after (arrow) receiving poly I:C versus saline via icv injection. BT data is expressed as hourly average. (B) Peak rise (Δ BT) in body temperature during 24 h in WT versus DKO mice following poly I:C versus saline via icv injection. (C) Effect of icv poly I:C versus saline administration on voluntary LMA in WT versus DKO mice. Movement data expressed as hourly sums. (D) Sum total of voluntary LMA during 12-h dark phase in WT versus DKO mice treated with poly I:C or saline via icv injection. (E) Body temperature in WT versus DKO mice before and after (arrow) receiving intraperitoneal poly I:C versus saline. BT expressed as hourly average. (F) Peak rise in body temperature (Δ BT) during 24 h in WT versus DKO mice following poly I:C or saline via ip injection. (G) Effect of ip poly I:C versus saline administration on voluntary LMA in WT versus DKO mice. LMA expressed as hourly sums. (H) Sum total of voluntary LMA during 12h-dark phase in WT versus DKO mice treated with poly I:C or saline via ip injection. Poly I:C or saline administered via icv or ip injection at ZT 9; arrows depict time of icv or ip injection. Shaded regions indicates dark phase. $^{**}p < 0.01$, and $^{***}p < 0.001$. Error bars represent SEM.

Figure S4.

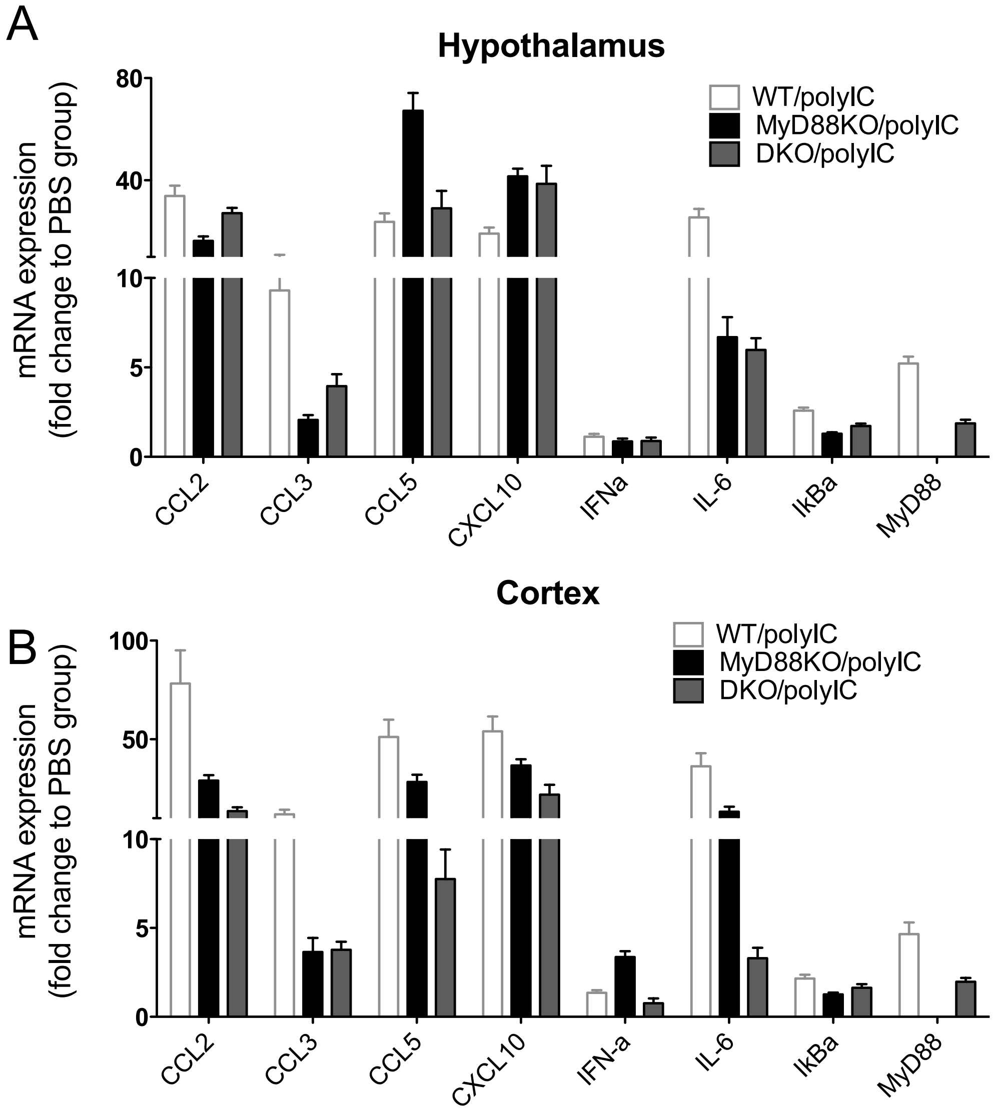

Figure S4, related to figure 3. Follow up analysis of expression of multiple inflammatory genes in WT, MyD88KO, and DKO mice. 6 h-post icv injection of saline or polyIC, brains were extensively flushed by transcardial perfusion with PBS before dissection of hypothalami and cortices. Fold change indicates relative quantification (RQ) of mRNA in poly IC-treated group vs. saline-treated group within each genotype. Error bars represent SEM. (A) Gene expression from microdissected hypothalamus. (B) Gene expression in dissected cortex.

Figure S5.

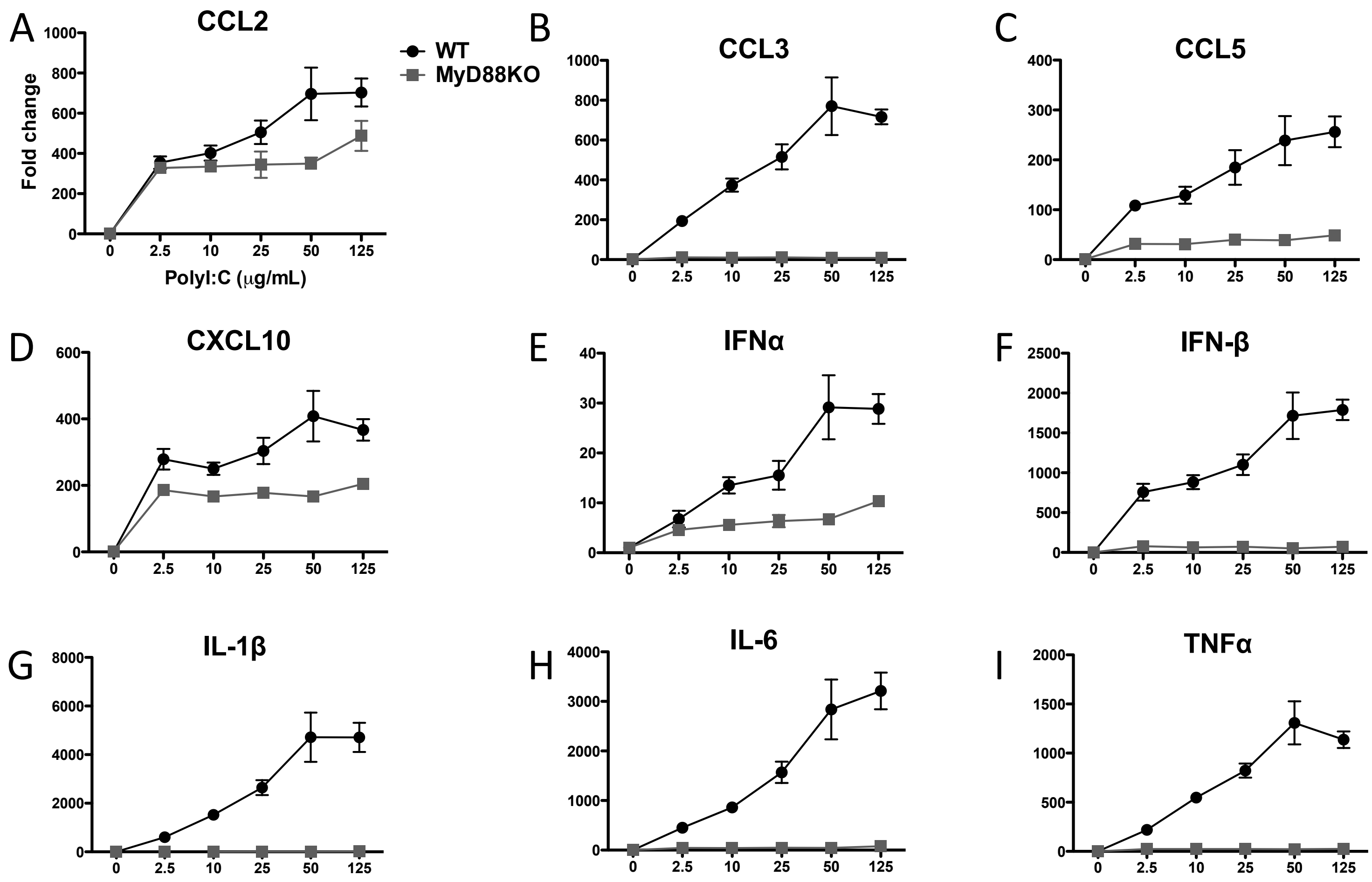

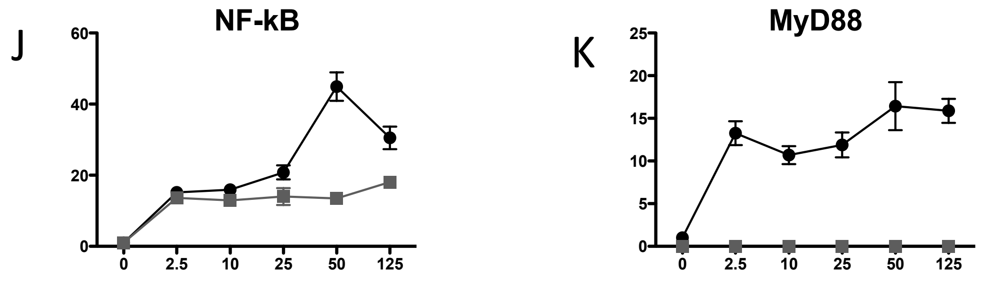

Figure S5, related to figure 6. Induction of gene expression of chemokines, type I IFNs and cytokines in mixed-glial cells was poly IC dose-dependent, but highly attenuated in MyD88KO mixed-glial cells. A-L. Primary cultures of mixed glial cells from WT and MyD88KO mice were stimulated with PBS or poly IC (2.5, 10, 25, 50, 125 μg/ml) for 6 h. RT-PCR was performed in these stimulated cells. Fold change indicates mRNA relative quantification (RQ) in poly IC-stimulated cells vs. PBS-stimulated cells within each genotype. Error bars represent SEM. Similar results were observed in at least two independent experiments.

Figure S6. Western Blot of cell extracts from mixed glia.

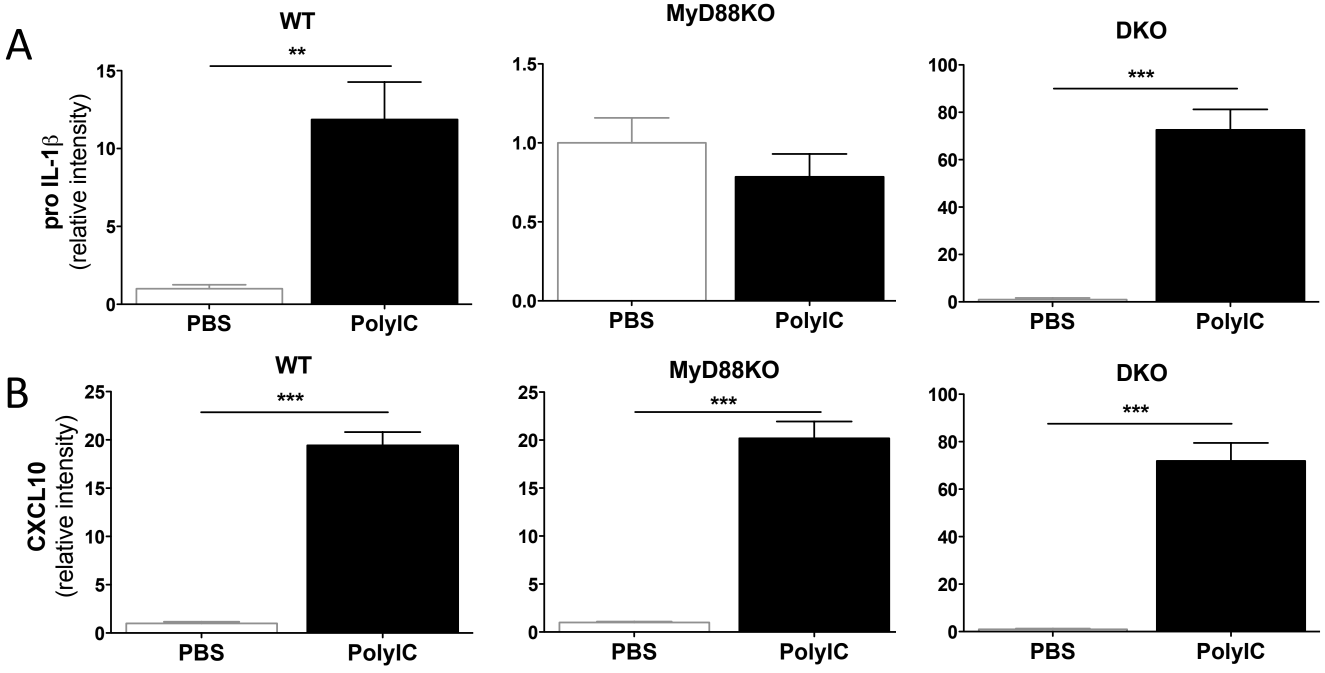

Figure S6, related to figure 6. Poly IC induced-protein synthesis of proIL-1b was highly attenuated in MyD88KO mixed-glial cells relative to WT or DKO cells.

Primary cultures of mixed-glial cells from WT, MyD88KO and DKO mice were stimulated with PBS or poly IC (50 μg/ml) for 6 h. Western blots were performed in protein extracted from the stimulated cells (n=4-5 individual plates for each genotype and condition). A. Relative intensity of proIL-1b or (B) CXCL10 bands relative to b-actin in poly IC-stimulated cells vs. PBS-stimulated cells is shown. Error bars represent SEM. Similar results were observed in at least two independent experiments. **$p < 0.01$ and ***$p < 0.001$.

Figure S7.

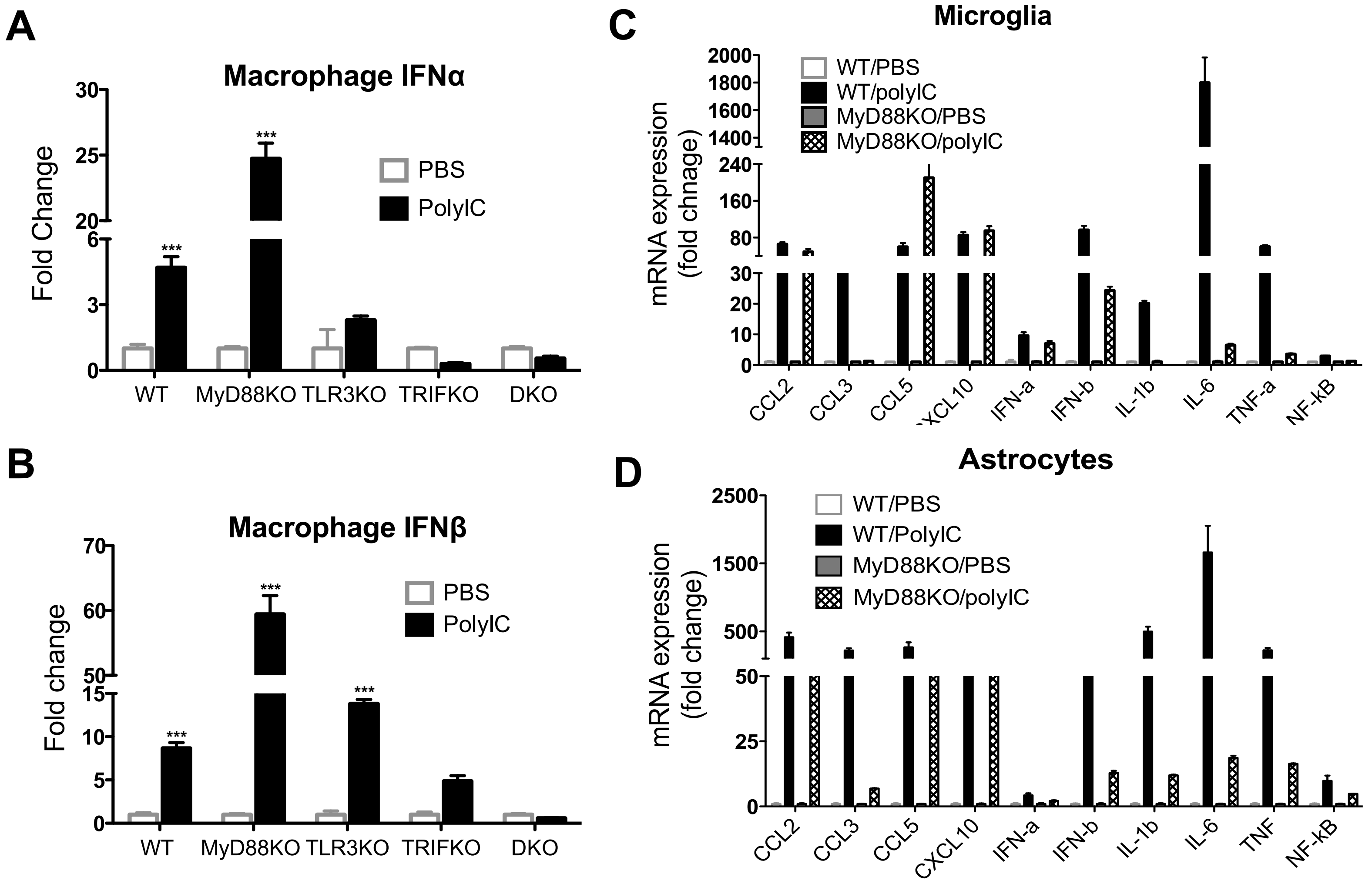

Figure S7, **related to figure 7. Poly IC induced-gene expression of chemokines, interferons and cytokines in purified cultured microglia, astrocytes, or peritoneal macrophages.**

A, B. Primary cultures of peritoneal macrophages from WT, MyD88KO, TLR3KO, TIRFKO and DKO mice were stimulated with PBS or poly IC (50 μg/ml) for 6 h. Fold change indicates relative quantification (RQ) of IFNa (A) or IFNb (B) mRNA in poly IC-stimulated cells vs. PBS-stimulated cells within each genotype. C, D. Primary cultures of highly enriched WT and MyD88KO microglia (C) or astrocytes (D) were stimulated with PBS or poly IC (50 μg/ml) for 6 h. Fold change indicates relative quantification (RQ) of mRNA in poly IC-stimulated cells vs. PBS-stimulated cells is shown. Error bars represent SEM. Similar results were observed in at least two independent experiments. *** $p<0.001$ relative to saline.

Supplemental Tables

Table S1. Food Intake and BW after icv Injection, Related to Figure 1

TABLE 3	Food Intake after icv treatment				Body Weight	
	4 Hour Intake		24 Hour Intake		% Change at 24 Hours	
Genotype	Saline	Poly(I:C)	Saline	Poly(I:C)	Saline	Poly(I:C)
WT (46)[b]	1.37±0.05	0.24±0.03***	4.28±0.08	2.16±0.09***	-0.26±.20	-6.88±0.36***
fWT (7)[c]	1.27±0.21	0.19±0.02***	4.27±0.14	2.13±0.09***	-0.07±0.34	-7.46±0.44***
TLR3KO (6)	1.41±0.13	0.34±0.05***	4.19±0.20	2.34±0.16***	-0.43±0.40	-4.30±0.72***
TRIFKO (5)	1.04±0.07	0.40±0.10***	3.91±0.15	2.39±0.18***	0.56±0.58	-3.11±0.71**
MDA5KO (6)	1.14±0.14	0.23±0.03***	4.40±0.20	2.30±0.18***	0.10±0.32	-5.70±0.51***
IPS-1KO (4)	0.96±0.03	0.27±0.02***	3.62±0.11	1.92±0.14***	-1.84±0.50	-7.81±0.33***
IL-1RKO (6)	1.21±0.13	0.26±0.07***	3.81±0.30	2.10±0.12***	-0.57±0.59	-4.31±0.32***
DKO (6)	1.04±0.08	0.34±0.05***	3.73±0.15	2.30±0.21***	-1.73±0.69	-7.18±1.1***
TLR2 KO (6)	1.33±0.19	0.15±0.03***	4.02±0.15	1.86±0.05***	-0.30±0.20	-6.93±0.64***
TLR4 KO (4)	1.03±0.10	0.25±0.03***	3.87±0.46	2.72±0.08*	-0.64±0.36	-3.97±0.31
TLR7 KO (7F)[c]	1.30±0.08	0.24±.05***	4.39±0.14	2.48±0.27***	0.75±0.71	-7.5±0.92***
TLR9 KO (5)	0.92±0.06	0.11±0.03***	3.91±0.18	1.84±0.07***	-0.08±.40	-8.13±0.88***
MyD88 KO (7)	1.17±0.07	0.96±0.06	3.59±0.11	3.29±0.17	0.26±0.51	-2.4±0.21
fMyD88KO (7)[c]	0.84±0.05	0.73±0.09	3.43±0.21	3.42±0.16	-0.38±0.74	-1.29±0.97
MyD88KOfl/fl Slco1c1^{CreER}(6)	1.11±.08	0.13±.03***	4.85±.18	2.1±0.29***	0.19±.66	-8.4±.16***

[a] Poly IC: 20 µg/mouse dissolved in 2 µl saline for icv injection.
[b] WT (46) was combined all WT control mice used in individual feeding studies.
[c] All feeding studies were performed in male mice but also in WT, MyD88 KO female mice (F).
TLR7 KO mice were female (F).
Values are means ± SE; (n), number of animals. *P < 0.05, ***P < 0.001 vs. saline group.

Table S2. Food Intake and BW After ip Injection, Related to Figure 1

TABLE 2	Food Intake after i.p. treatment				Body Weight	
	4 Hour Intake		24 Hour Intake		% Change at 24 Hours	
Genotype	Saline	Poly(I:C)	Saline	Poly(I:C)	Saline	Poly(I:C)
WT (39)[b]	1.23±0.06	0.38±0.03***	4.15±0.07	2.69±0.10***	-0.46±0.26	-3.74±0.27***
WT (7, F)[c]	1.40±0.05	0.33±0.05***	4.48±0.10	3.20±0.18***	0.37±0.55	-2.96±0.84**
TLR3KO (6)	1.40±0.04	1.56±0.10	4.96±0.14	4.72±0.14	0.20±0.47	0.88±0.66
TRIFKO (8)	1.17±0.07	0.94±0.13	4.06±0.18	4.01±0.19	0.29±0.45	-1.00±0.82
MDA5KO (6)	1.15±0.11	0.31±0.06***	4.21±0.28	2.49±0.23***	-0.34±0.43	-4.77±0.83***
IPS-1KO (4)	1.07±0.12	0.17±0.02***	3.88±0.16	1.88±0.11***	-1.85±0.44	-6.20±0.51***
DKO (7)	1.19±0.07	1.08±0.12	3.87±0.12	3.99±0.18	-1.27±0.31	-1.30±0.33
TLR2 KO (6)	2.84±0.52	0.89±0.34*	4.23±0.14	2.02±0.14***	-0.17±0.44	-3.81±0.40***
TLR4 KO (8)	1.41±0.06	0.55±0.06***	4.43±0.10	3.17±0.28**	-0.55±0.26	-3.57±0.43***
TLR7 KO (7, F)[c]	1.18±0.05	0.24±0.03***	4.23±0.14	3.00±0.19***	-0.87±0.76	-3.18±0.42*
IL-1RKO (7)	1.22±0.11	0.75±0.05**	3.89±0.15	2.93±0.30*	-1.24±0.41	-2.01±0.67
MyD88KO (8)	1.40±0.07	0.78±0.04***	3.94±0.15	2.94±0.08***	-0.24±0.53	-3.81±0.40***
MyD88KO(8,F)[c]	1.06±0.09	0.30±0.03***	3.75±0.19	2.99±0.20*	0.02±0.61	-3.61±0.66***

[a] Poly IC: Poly IC: 10 mg/kg dissolved in 100µl saline for ip injection.

[b] WT (37) was combined all WT control mice used in individual feeding studies.

[c] All feeding studies were performed in male mice but also in MyD88 KO female mice (MyD88 KO, F). TLR7 KO mice were female (F).

Values are means ± SE; n, number of animals. *$P < 0.05$, **$P < 0.01$, ***$P < 0.001$ vs. saline group.

Supplemental Experimental Procedures

Mice

Knockout mice of TLR2, TLR3, TLR4, MDA5, IL-1R, MyD88, TRIF, IPS-1 and age-sex-matched C57BL/6J wild type (WT) mice were purchased from The Jackson Laboratory (Bar Harbor, ME). TLR9 KO mice were provided by Dr. Mary Stenzel-Poore. Double knockout (DKO) mice of TRIF/IPS-1 were generated by intercrossing TRIFKO and IPS-1KO mice as described previously (Kumar et al., 2008; Menasria et al., 2013). KO and DKO mice were genotyped using standard protocols from The Jackson Laboratory. All mice were housed in rooms with controlled temperature (25 ± 2^0C) and illumination (12 h light/12 h dark) and provided *ad libitum* access to water and food (Purina rodent diet 5001; Purina Mills, St. Louis, MO, USA) unless otherwise stated, and were allowed to acclimate for at least 7 d before procedures. Adult mice between 8-12 wk of age were used for *in vivo* experiments, and newborn mice (postnatal day 1 to 3) generated from our own breeding colonies were used for *in vitro* experiments. All studies were conducted according to the National Institutes of Health Guide for the Care and Use of Laboratory Animals and approved by the Institutional Animal Care and Use Committee of Oregon Health & Science University.

For *in vivo* experiments, one day before each experiment commenced, the animals were weighed and divided into treatment groups such that the mean body weights of each group were similar. During the experiments, food intake and body weight were measured at the same time each day, unless otherwise noted.

Intracerebroventricular and Intraperitoneal Injections

Under isofluorane anesthesia, 26-gauge lateral ventricle cannulas (PlasticsOne, Roanoke, VA, USA) were placed in mice using a stereotactic alignment instrument (Kopf, Tujunga, CA, USA) at the following coordinates relative to bregma: 1.0 mm X, -0.5 mm Y and -2.25 mm Z. Mice were then individually housed and allowed to recover from surgery for at least 7 days. Poly I:C (Sigma, St. Louis, MO, USA) was dissolved in 0.9% saline according to the manufacturer's instruction. 0.9% saline or 20 μg of poly I:C (per mouse) was given in 2 μl total volume through intracerebroventricular (icv) injection. For peripheral treatment, 0.9% saline or poly I:C at 10 mg/kg body weight was injected intraperitoneally (ip).

Body Temperature and Locomotor Activity Measurement

Body temperature (BT) and voluntary home cage locomotor activity (LMA) were measured using a MiniMitter system (MiniMitter, Bend, OR, USA) as described previously (Braun et al., 2012; Grossberg et al., 2011). Briefly, under isofluorane anesthesia, transponders for sensing BT and LMA were implanted adjacent to the abdominal aorta in the retroperitoneal space. Mice were allowed to acclimate for at least 7 days before BT and movement in x-, y- and z-axes were recorded in 5- minute intervals (Vital View, MiniMitter). Δ BT was calculated in B and D using formula $\Delta BT = BT_{post\text{-}injection} - BT_{pre\text{-}injection}$ at a same ZT point during the 48h period of before and after treatment.

Nocturnal Feeding Studies

All feeding studies were performed at night, as described previously (Zhu et al., 2012). Mice were individually housed for conditioning for at least 7 days. 2 days before treatment, mice were placed in clean individual cages with measured amounts of food. Baseline body weight change and 24 h food intake were obtained on two consecutive days. On the experimental day, 3h before lights off, mice were administered poly I:C at 20 μg /mouse (icv) or 10 mg/kg body weight (ip), respectively, and pre-weighed food pellets were placed into each cage at 5:00 PM. Food was weighed at 4 time points (2 h, 4 h, 16 h and 24 h). Body weights were measured at 2 time points (16 h and 24 h). Care was taken to minimize stress and light exposure to the animals during the nighttime of food measurements.

Tissue Collection

For RT-PCR analysis in tissues, 6 h after icv injection, mice were deeply anesthetized using a ketamine cocktail and sacrificed by trans-cardiac perfusion (≥50 ml) with PBS to remove intravascular blood. Brains and inter-scapular brown adipose tissue (BAT) were immediately removed. Hypothalamic and cortical blocks were dissected. Hypothalami, cortices and BAT were snap frozen and stored in -80^0C until analysis. For immunohistochemistry (IHC) analysis of lymphocytes in brain tissue, after two icv injections (two doses/mouse within 24 h), mice were perfused by PBS

followed by 4% paraformaldehyde (PFA) for tissue fixation. Brains were post-fixed in 4% PFA for overnight and cryoprotected in 20% sucrose for 24 h at 4^0C before being stored at -80^0C until IHC analysis.

Primary Culture

Mixed-Cultures: Primary mixed-cultures of microglia and astrocytes were prepared from neonatal mouse cortices as described previously (Witting and Moller, 2011) with modifications. Briefly, under sterile conditions cerebral brains from newborn mice were dissected, freed of the meninges, and were kept on ice before digestion using papain (Worthington, Biochemical Corporation). Dissociated mixed-cells in complete medium (DMEM low glucose with L-glutamine, 10% FBS and 1% penicillin/streptomycin) were seeded in 75-cm^2 flasks or 100-mm dishes. Primary mixed-cultures were incubated for 14-16 days by feeding complete medium twice a week so that only glial cells remained. For RT-PCR in mixed-cultures, cells were harvested and re-plated into 6-well plates (Falcon; BD Bioscience) at 1×10^6 cells/well with complete medium for 1-2 days before stimulation. For Western blots in mixed-cultures, cells were cultured in 100-mm dishes for 14-16 days until stimulation.

Highly-Enriched Microglia: As previously described (Chen et al., 2015; Witting and Moller, 2011), highly-enriched microglia were isolated from mixed-cultures by shaking flasks at 200 rpm at 37^0C for 2 h in an incubator-shaker. Cells were re-plated into 6-well plates at 5×10^5/well and maintained with complete medium for overnight before stimulation. More than 99% of these isolated cells were confirmed as microglia by ICC Iba1 staining.

Highly-Enriched Astrocytes: We utilized cultures containing L-leucine methyl ester (LME, Sigma, St. Louis, MO, USA) in mixed-cultures (Chen et al., 2015; Saura, 2007). Briefly, mixed-cells were harvested from newborn mouse cortices and seeded into 6-well plates at $1.2\text{-}1.5 \times 10^6$ cells/well with complete medium described above. 72 h after seeding, LME at 1 nM (final concentration) was applied in complete medium for 6 days before stimulation (medium with LME was changed once during 6-day culture period). More than 99% of these cells stained positive for GFAP by ICC, confirming their identity as astrocytes.

Peritoneal Macrophages: Mouse peritoneal macrophages were harvested after elicitation with 3% Brewer thioglycollate medium for 4 days as described previously (Zhang et al., 2008). Cells were re-plated into 6-well plates at $5\text{-}10 \times 10^6$ cells/well and maintained with DMEM/F12-10 medium overnight before stimulation.

Cell Stimulation and Sample Collection

For RT-PCR in mixed-cultures, cells were stimulated with PBS or poly I:C at 50 μg/ml for 6 h. For RT-PCR or for Western blots, cells were stimulated with PBS or poly I:C at 50 μg/ml for 6 h. During sample collection, culture supernatants were first collected, and adherent cells were washed and lysed using cell lysis buffer (RT-PCR, RLT buffer, Qiagen Inc. Valencia, CA; Western blots, Cell Signaling, Danvers, MA, USA). RNA preparation and protein extraction were performed as described previously (Braun et al., 2012; Zhu et al., 2012). RNA and protein samples were stored in -80^0C until analysis.

Quantitative RT-PCR

Real-time quantitative PCR (RT-PCR) was performed as described previously (Zhu et al., 2012). RNA was extracted from tissues or cells using RNeasy kits (Qiagen Inc., Valencia, CA). Reverse transcription and quantitative PCR reagents were obtained from Life Technologies (Carlsbad, CA). 18S, β-actin or GAPDH cDNA were used as endogenous control. Gene expression is reported as mRNA fold change relative to saline or PBS treated group within genotype of mice or cells using the 2^{-DDCt} method. Statistical analyses were performed on the ΔCt values.

Western Blot

Protein synthesis of inflammatory cytokines in mixed-cultures was measured by Western blots. 35 μg per lane (IL-1β) or 5 μg per lane (CXCL10) of total protein extracted from mixed-cultures was run on Novex 10-20% Tris-Glycine gels (Life Technologies) at 120V. Gels were transferred to Immobilon-FL membranes (Millipore) and blocked with 5% BSA for 1 h. Membranes were incubated o/n with β-actin mouse mAB 1:4000 (Cell Signaling, #3700) and either IL-1β Rabbit mAB 1:1000 (Cell Signaling, #12426) or Anti-IP10 (CXCL10) Rabbit 1:1000 (Abcam, #ab9938). After 4x washing with TBST, membranes were incubated for 1 h with DyLight 680 Anti-rabbit and 800 Anti-mouse 1:15000 ea (Cell Signaling), then visualized.

ELISA

IL-6 or IL-1β in culture supernatants were measured using ELISA kits according to manufacturing instructions (IL-6 kit was purchased from BD Biosciences; IL-1β kit was purchased from eBioscience).

Immunohistochemistry

Primary cultured mixed glial cells and highly-enriched microglia and astrocytes were cultured directly on poly-D-lysine/laminin coated glass coverslips (Corning Biocoat, German) in 24-well plates at 1-2 × 10^5 cells/ml with complete medium for 24h. Cells were washed with PBS and fixed with 4% paraformaldehyde for 20 min on ice. Using a similar protocol as described previously (Zhu et al., 2012). For brain histochemistry, mice were sacrificed and brains processed as described previously (Scarlett et al., 2007).

Cultured glia: Immunofluorescent staining was processed with primary antibodies GFAP diluted 1:1000 (MAb360, Millipore) and Iba1 diluted 1:500 (Wako) and secondary fluorescent antibodies (goat anti-mouse Alexa Flour 594 with 1:500 dilution or goat anti-rabbit 488 with 1:500 dilution; Invitrogen) for visualization. In the negative controls, primary antibodies were omitted. Cell nuclei were labeled with DAPI at 1: 25,000 dilution for 5 min.

Hypothalamic CD45 Cells: IHC in brain tissue was performed as described previously (Zhu et al., 2012). Briefly, four series of 30-μm coronal sections were cut and collected throughout the hypothalamus. One series of hypothalamic sections was processed for CD45 IHC. After washing and blocking, floating sections were incubated with CD45 primary antibody (rat anti-mouse CD45, 1:1000; BD Biosciences) for overnight at 4^0C, followed by incubation with secondary antibody conjugated with Alexa Flour 555 (1:500; Invitrogen) for visualization. Sections were viewed and the number of CD45 immuno-fluorescence positive cells was manually counted under a fluorescent microscope (model 4000 DM, Leica Microsystems).

POMC neurons: For POMC neuronal staining, we used anti-POMC (rabbit antibody, 1:5000 dilution, Phoenix Pharmaceuticals) and anti-cFos (goat antibody, 1:25000 dilution, Santa Cruz). Secondary antibodies were donkey anti-rabbit (Alexa Flour 594, 1:500, red for POMC) and donkey anti- goat (Alexa Flour 488, 1:500, green for cFos, Invitrogen). Rostral-caudal distribution of sections for each hypothalamus was matched prior to blinded cell counting. Confocal photomicrographs were taken using a Zeiss LSM700 confocal microscope (Carl Zeiss, Oberkochen, Germany) under identical microscope settings. Images were processed using NIH ImageJ software.

Statistical Analysis

All data are expressed as mean ± standard error of the mean (SEM) for each group. Statistical analyses were performed using the unpaired Student's t-Test or ANOVA followed by Bonferroni posttests using GraphPad Prism 5 (La Jolla, CA). $p<0.05$ was considered statistically significant.

Supplemental References

Braun, T.P., Grossberg, A.J., Veleva-Rotse, B.O., Maxson, J.E., Szumowski, M., Barnes, A.P., and Marks, D.L. (2012). Expression of myeloid differentiation factor 88 in neurons is not requisite for the induction of sickness behavior by interleukin-1beta. J Neuroinflammation *9*, 229.

Chen, S.H., Oyarzabal, E.A., Sung, Y.F., Chu, C.H., Wang, Q., Chen, S.L., Lu, R.B., and Hong, J.S. (2015). Microglial regulation of immunological and neuroprotective functions of astroglia. Glia *63*, 118-131.

Grossberg, A.J., Zhu, X., Leinninger, G.M., Levasseur, P.R., Braun, T.P., Myers, M.G., Jr., and Marks, D.L. (2011). Inflammation-induced lethargy is mediated by suppression of orexin neuron activity. J Neurosci *31*, 11376-11386.

Kumar, H., Koyama, S., Ishii, K.J., Kawai, T., and Akira, S. (2008). Cutting edge: cooperation of IPS-1- and TRIF-dependent pathways in poly IC-enhanced antibody production and cytotoxic T cell responses. J Immunol *180*, 683-687.

Menasria, R., Boivin, N., Lebel, M., Piret, J., Gosselin, J., and Boivin, G. (2013). Both TRIF and IPS-1 adaptor proteins contribute to the cerebral innate immune response against herpes simplex virus 1 infection. J Virol *87*, 7301-7308.

Saura, J. (2007). Microglial cells in astroglial cultures: a cautionary note. J Neuroinflammation *4*, 26.
Witting, A., and Moller, T. (2011). Microglia cell culture: a primer for the novice. Methods Mol Biol *758*, 49-66.

Zhang, X., Goncalves, R., and Mosser, D.M. (2008). The isolation and characterization of murine macrophages. Curr Protoc Immunol *Chapter 14*, Unit 14 11.

Zhu, X., Krasnow, S.M., Roth-Carter, Q.R., Levasseur, P.R., Braun, T.P., Grossberg, A.J., and Marks, D.L. (2012). Hypothalamic signaling in anorexia induced by indispensable amino acid deficiency. Am J Physiol Endocrinol Metab *303*, E1446-1458.
